# Beyond reverse transcription: molecular mechanisms and emerging paradigms in retroviral replication

**DOI:** 10.1093/femsre/fuaf066

**Published:** 2025-12-26

**Authors:** Mohammad Abdullah Jehad, Lizna M Ali, Vineeta N Pillai, Suresha G Prabhu, Farah Mustafa, Tahir A Rizvi

**Affiliations:** Department of Microbiology and Immunology, College of Medicine and Health Sciences (CMHS), United Arab Emirates University, Al Ain, United Arab Emirates; Department of Microbiology and Immunology, College of Medicine and Health Sciences (CMHS), United Arab Emirates University, Al Ain, United Arab Emirates; Department of Microbiology and Immunology, College of Medicine and Health Sciences (CMHS), United Arab Emirates University, Al Ain, United Arab Emirates; Department of Microbiology and Immunology, College of Medicine and Health Sciences (CMHS), United Arab Emirates University, Al Ain, United Arab Emirates; Department of Biochemistry and Molecular Biology, College of Medicine and Health Sciences (CMHS), United Arab Emirates University, Al Ain, United Arab Emirates; Zayed bin Sultan Center for Health Sciences (ZCHS), United Arab Emirates University, Al Ain, United Arab Emirates; Department of Microbiology and Immunology, College of Medicine and Health Sciences (CMHS), United Arab Emirates University, Al Ain, United Arab Emirates; Zayed bin Sultan Center for Health Sciences (ZCHS), United Arab Emirates University, Al Ain, United Arab Emirates

**Keywords:** retroviruses, classification and nomenclature, reverse transcription, integration, transcription, nuclear export, translation, RNA packaging, virion assembly, budding and maturation

## Abstract

Retroviruses are exclusive group of positive-sense RNA viruses defined by their ability to reverse transcribe their RNA genome and integrate it into the host’s chromosomal DNA. This distinctive replication strategy enables persistent infection and has profoundly shaped our understanding of molecular biology, gene regulation, and evolution. Retroviruses have contributed to landmark discoveries, including the identification of oncogenes, mechanisms of transcriptional control, and the development of gene therapy vectors. This review provides an updated overview of retroviral molecular biology, emphasizing the coordinated steps of the viral life cycle and emerging insights that are reshaping classical models. It explores virion structure, genome organization, and the interplay of *cis*-acting sequences and *trans*-acting factors that govern replication. Special focus is given to recent advances in understanding nuclear trafficking of capsids, spatial dynamics of reverse transcription and integration leading to provirus formation, RNA nuclear export, and selective genome packaging. The structural and functional roles of viral proteins, particularly Gag, are discussed in the context of assembly and maturation. By integrating foundational concepts with new discoveries, this review highlights the molecular sophistication of retroviral replication and identifies outstanding questions that guide future research, with implications extending to antiviral strategies, gene therapy, cancer biology, and evolution.

## Teaser

This review unites emerging discoveries with classical understanding on how retroviruses replicate and interact with their hosts, offering fresh insights into molecular virology and host cell biology.

## Introduction

Retroviruses belong to a distinguished class of positive-sense RNA viruses. Despite possessing all the canonical features of a classical mRNA, such as a methylated cap at the 5′ end and a polyadenylated tail at the 3′ end, they are not immediately translated into proteins by the host cell machinery like other positive-sense RNA viruses. Instead, they adopt a unique replication strategy involving reverse transcription and genomic integration, processes which undoubtedly have been instrumental in advancing our understanding of today’s molecular biology. Although a singular recent report suggests the possibility of direct translation of the retroviral genome (Köppke et al. 2024), the prevailing consensus remains that reverse transcription, and genomic integration must occur and translation happens only from transcripts generated following proviral integration.

Retroviruses can infect a broad spectrum of vertebrate species, particularly mammals, and include several clinically and biologically important members (Goff 2013, Skalka 2018). Some retroviruses are responsible for causing immunodeficiency in humans and animals, such as human, feline, simian, and bovine immunodeficiency viruses (HIV, FIV, SIV, and BIV) and Mason–Pfizer monkey virus (MPMV); neoplastic diseases caused by Rous sarcoma virus (RSV), avian leukosis virus (ALV), human T-lymphotropic virus type 1 (HTLV-1), murine, feline, and bovine leukemia viruses (MLV, FLV, and BLV), and mouse mammary tumor virus (MMTV; Goff 2013, Skalka 2018). The first retrovirus to be identified at the beginning of the 20th century was ALV, the causative agent of leukemia in chickens (Ellermann and Bang 1908). Later, Rous (1911) demonstrated that sarcomas could be transmitted in chickens through cell-free filtrates, leading to the identification of RSV. HTLV-1, the only known human retrovirus to date that causes malignancy in humans, was discovered in the latter part of the 20th century (Poiesz et al. 1980, Yoshida et al. 1982), and soon thereafter, HIV-1 was identified as the causative agent of the acquired immunodeficiency syndrome (AIDS) (Barré-Sinoussi et al. 1983, Gallo et al. 1984).

Since their discovery, retroviruses have been instrumental in several biological discoveries, for example cell transformation, both viral and cellular oncogenes, reverse transcription, and integration into the host genome. More recently, a deeper understanding of retroviral life-cycle-facilitated cDNA cloning and the refinement of retroviral vectors for human gene therapy (Telesnitsky and Goff 1997, Wang et al. 2025). As mentioned above, in contrast to the positive-sense RNA viruses, retroviral genomes exploit virally encoded reverse transcriptase (RT) enzyme to reverse transcribe RNA into a viral DNA intermediate, which is then inserted into the host cell chromosome, a process that is also mediated by the another virally encoded enzyme, integrase (IN; Telesnitsky and Goff 1997). Retroviruses ensure the persistence of their life cycle and establish a permanent presence within the host’s hereditary material through integration of their genetic material into the host genome.

Due to their unique ability to integrate into a host’s cell DNA, retroviruses have also played a significant role in genetic diversity, human health, and evolutionary processes, profoundly influencing the evolutionary trajectories of numerous organisms (Jern and Coffin 2008, Moelling and Broecker 2019). The integrated remnants of ancient retroviral infections, called endogenous retroviruses, are widespread in the eukaryotic genomes, integral to the creation of their genomes and contributing to their genetic diversity (Katzourakis and Gifford 2010, Mager and Stoye 2015, Johnson 2019). Beyond their evolutionary significance, retroviruses are also pivotal in medical research. They have been instrumental in understanding viral replication, gene regulation, and the development of gene therapies, as well as therapies for various virally induced diseases, such as cancer and AIDS. This dual role of retroviruses in both evolution and medicine underscores their profound importance in the natural world and human health. Undoubtedly, the discovery of HIV-1 greatly advanced the field of retroviruses, but the initial observations/discoveries made significant contributions toward our current understanding of tumorigenesis and molecular biology (Baltimore 1970, Temin and Mizutani 1970, Stehelin et al. 1976).

A deeper understanding of retroviral biology led to the dawn of recombinant DNA technology, which has heralded the possibility of using genes for the cure of innumerable human diseases into the dominion of reality. The prospect of using gene therapy to treat, prevent, and control human diseases, such as cardiopulmonary disorders, cancer, and diabetes, has revolutionized the field of biomedical research and could potentially save and improve the quality of millions of lives (Song et al. 2004, Ly et al. 2008, Hu et al. 2011, Chellappan et al. 2018, Anguela and High 2019, Collins et al. 2021). Toward this end, retroviruses have served as highly useful tools, especially in the development of gene therapy vectors for the delivery of therapeutic genes into host cells, revolutionizing molecular medicine. This progress stemmed from detailed studies on retroviral genome replication and its gRNA packaging into the assembling virion. The packaging of retroviral gRNA occurs concomitantly with viral assembly, which depends on the virus type, and takes place either at the plasma membrane (e.g. HIV-1 and FIV) or in the cytoplasm (e.g. MPMV and MMTV), followed by their budding from the infected cell (Goff 2013, Chameettachal et al. 2023).

This review provides a comprehensive overview of retroviral replication cycle, tracing each step, starting from entry, reverse transcription, integration, transcription, and viral gene expression to steps of virion assembly. Special attention is given to the unique enzymatic processes that set retroviruses apart from other viruses, such as reverse transcription and integration, as well as the dynamic interplay between viral and host factors that govern successful replication. It integrates classical paradigms with recent discoveries, we provide a comprehensive perspective on the RNA-based mechanisms that govern retroviral replication, thereby offering novel targets for therapeutic interventions. Furthermore, by synthesizing decades of foundational work with recent mechanistic insights, this review highlights how these discoveries continue to shape our understanding of the fundamentals of gene expression and their application toward the development of antiviral therapeutics against retrovirus-associated pathologies, as well as a better understanding of the molecular basis of diseases, especially cancer.

## Classification of retroviruses

**Figure 1.**
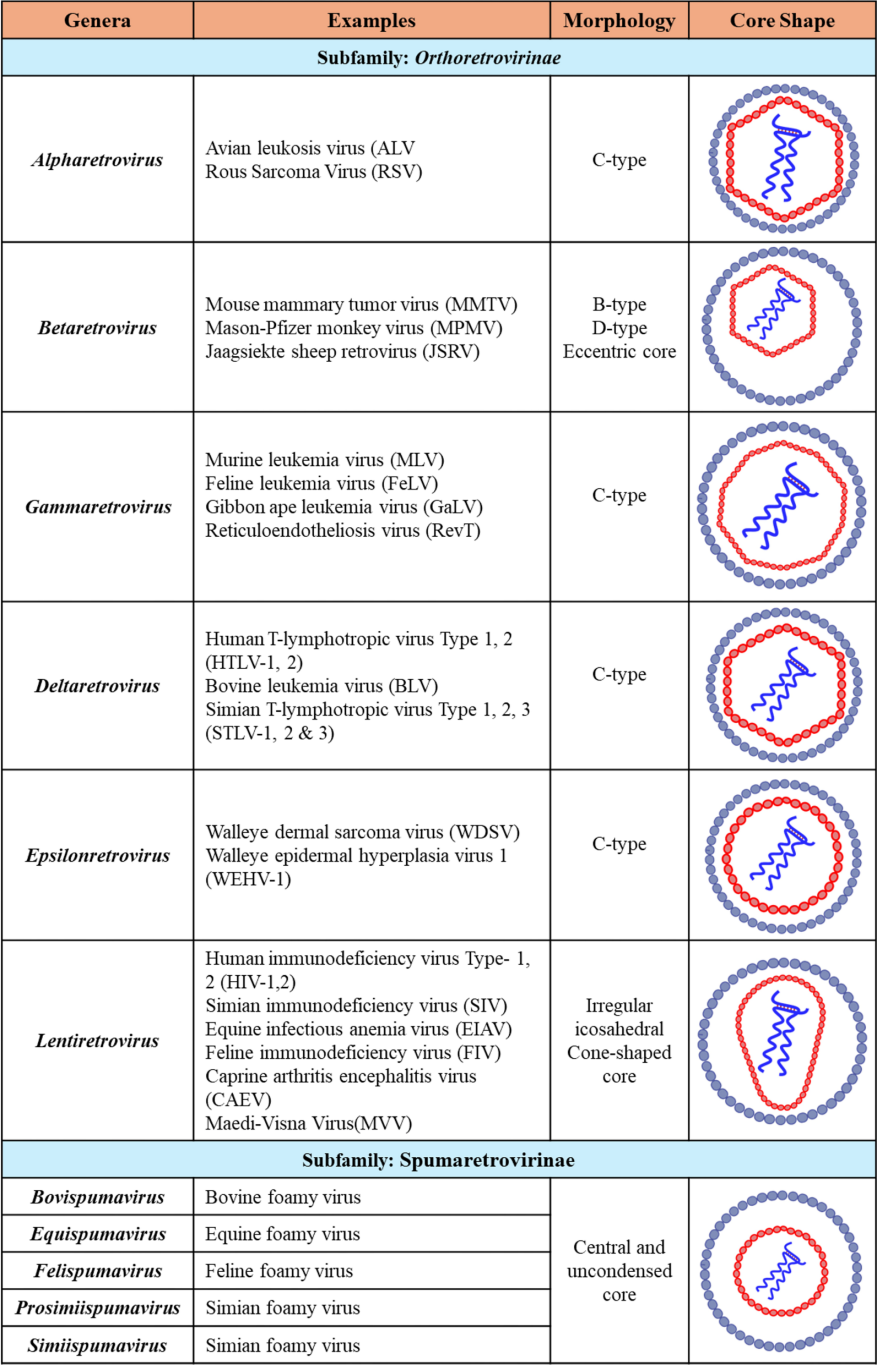
Morphology of core across retroviral genera. Illustration of the distinct mature core morphological types, characteristic of different retroviruses accompanied by representative examples from each group. Figure made in BioRender.com.

Retroviruses are a large and diverse group of enveloped positive-sense RNA viruses belonging to the family *Retroviridae*, members of which are found in a wide variety of vertebrate hosts (Coffin 1997). Retroviruses were initially classified based on the virion core morphology, as observed through electron microscopy. Based on this classification, the term “A-type” refers to viruses that form a thick shell with hollow, electron-lucent internal structures with a distinctive morphology. “B-type” viruses contain an inner core, which is rounded but eccentrically positioned. “C-type” viruses contain a central, symmetrically positioned, spherical inner core, type-D viruses have a cylindrical/bar-shaped core, and lentiviruses have an irregular icosahedral cone-shaped core (Fig. 1).

**Figure 2.**
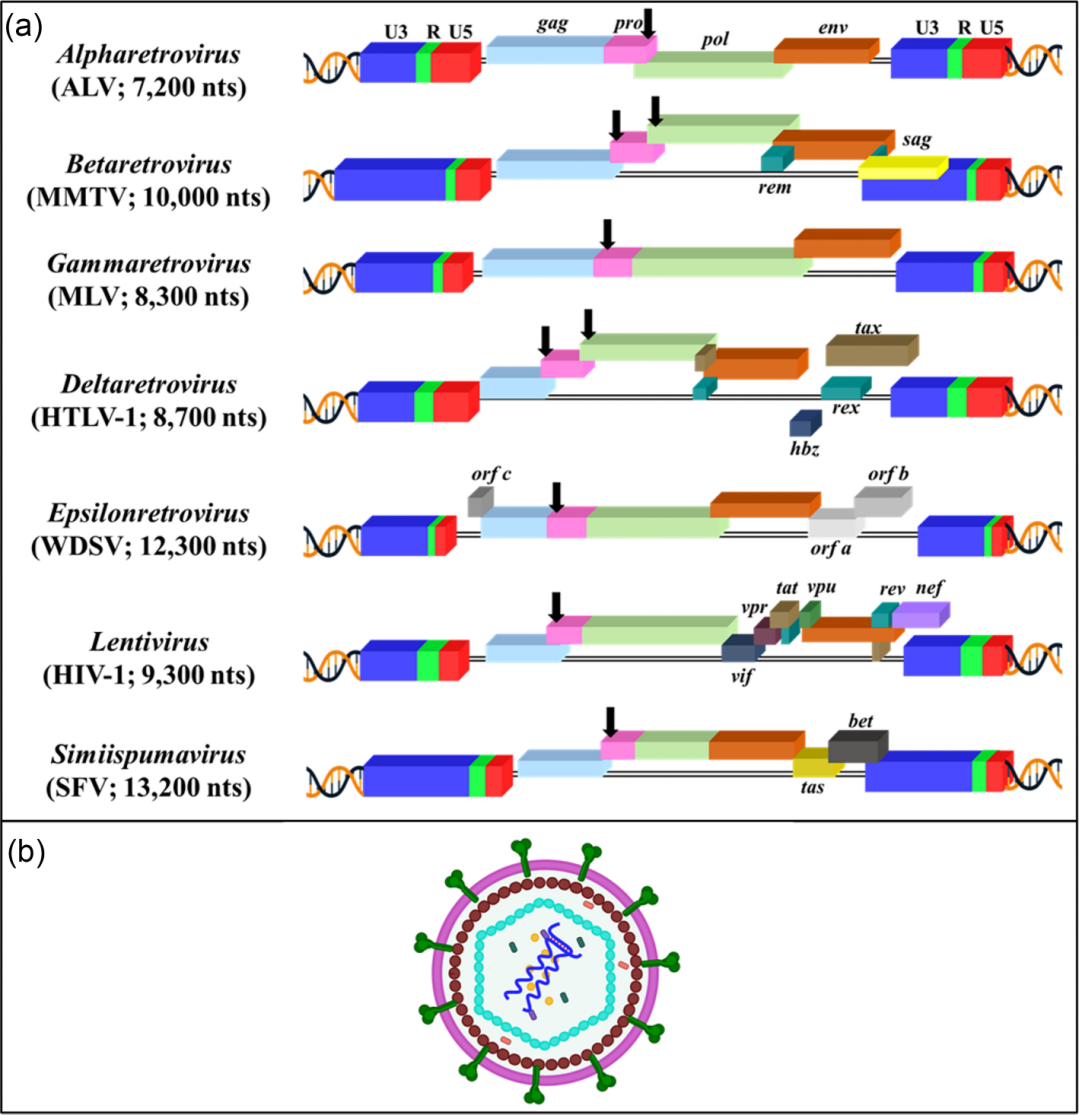
Schematic representation of (a) proviral genome organization across retroviral genera, and (b) a mature retroviral particle showing a dimeric genome inside the core. Representative viruses include: ALV, avian leukosis virus; MMTV, mouse mammary tumor virus; MLV, murine leukemia virus; HTLV-1, human T-lymphotropic virus type 1; WDSV, Walleye dermal sarcoma virus; HIV-1, human immunodeficiency virus type 1; and SFV, simian foamy virus. Black vertical arrows indicate the sites of ribosomal frameshifting within each proviral genome. nts: Nucleotides. Figure made in BioRender.com.

Based on the recent classification of the International Committee on Taxonomy of Viruses, the *Retroviridae* family has been divided into two subfamilies: *Orthoretrovirinae* and *Spumaretrovirinae*, encompassing a total of 11 genera (Coffin et al. 2021). The *Orthoretrovirinae* subfamily includes six genera: *Alpharetroviruses, Betaretroviruses, Deltaretroviruses, Epsilonretroviruses, Gammaretroviruses*, and *Lentiviruses*. The *Spumaretrovirinae* subfamily consists of five genera: *Bovispumavirus, Equispumavirus, Felispumavirus, Prosimiispumavirus*, and *Simiispumavirus*. Most retroviruses are classified as simple retroviruses, encoding essential gene products, such as group-specific antigen (Gag), protease (Pro), polymerase (Pol), and envelope (Env) (Fig. 2A). In contrast to this, *deltaretroviruses, epsilonretroviruses, lentiviruses*, and *spumaviruses* are classified as complex retroviruses. These complex retroviruses not only encode the core gene products but also encode various regulatory and accessory proteins translated from singly, doubly, or multiply spliced mRNAs, which are crucial for different functions during their replication and infection of the host cell, and survival within the host (Fig. 2A; Table 1).

**Table 1.** Regulatory and accessory proteins of complex retroviruses.

Name	Expressed mRNAs	Virus(es)/nature of protein	Function(s)	Key references
*Trans*-activator of transcription (Tat)	Doubly spliced	Lentiviruses/regulatory	Activation of viral promoter, transcript initiation, and elongation.	Berkhout et al. (1989), Feinberg et al. (1991)
*Trans*-activator of transcription (Tax)	Doubly spliced	HTLV/BLV/regulatory	Activation of viral promoter, transcript initiation, and elongation.	Sodroski et al. (1984), Cann et al. (1985), Felber et al. (1985), Fujisawa et al. (1986)
Regulator of virion (Rev)	Doubly spliced	Lentiviruses/regulatory	Nuclear export of partially and unspliced messages by binding to Rev responsive element (RRE).	Malim et al. (1989), Bai et al. (2014)
Regulator of virion (Rex)	Doubly spliced	HTLV/BLV/regulatory	Nuclear export of singly spliced and unspliced messages by binding to Rex responsive element (RexRE).	Kiyokawa et al. (1985), Inoue et al. (1987), Seiki et al. (1988), Dokhelar et al. (1989), Felber et al. (1989), Unge et al. (1991)
Viral infectivity factor (Vif)	Singly spliced	All lentiviruses except for EIAV/Accessory	Essential for viral infectivity by counteracting antiviral effects of host APOBEC3 that inhibit viral replication.	Sheehy et al. (2002), Larue et al. (2010)
Negative regulatory factor (Nef)	Doubly spliced	Lentiviruses/accessory	Downregulates CD4 and MHC I from the cell surface to prevent superinfection and enhance virion release, as well as avoid detection by cytotoxic T cells, respectively; increases virion infectivity by excluding host restriction factor SERINC3/5 from the virus particles; promotes antiapoptotic (Fas/TNF) and survival (Erk MAPK) signals to enhance persistence of infected cells, etc. Not essential for viral replication *in vitro*, but Nef-defective HIV-1 virions result in slow/no progression to AIDS.	Kirchhoff et al. (1995), Roeth and Collins (2006), Foster and Garcia (2008)
Viral protein U (Vpu)	Singly spliced	Lentiviruses (present only in HIV-1 and SIV)/accessory	Promotes viral release by interacting with the host factor BST-2/Tetherin.	Strebel et al. (1988), Terwilliger et al. (1989), Neil et al. (2008), Van Damme et al. (2008)
Viral protein R (Vpr)	Singly spliced	Lentiviruses (conserved across human and all primate lentiviruses)/accessory	*Trans*-activation of LTR, nuclear import of the preintegration complex, apoptosis, cell cycle arrest, and DNA damage response activation.	Roshal et al. (2003), Zimmerman et al. (2004, 2006), Lai et al. (2005), Belzile et al. (2010)
Viral protein X (Vpx)	Singly spliced	Lentiviruses such as HIV-2 and SIV but not HIV-1/accessory	Facilitates nuclear import of the preintegration complex and is essential for infecting nondividing cells like macrophages and dendritic cells. Cellular target for Vpx is SAMHD1.	Guyader et al. (1987, 1989), Yu et al. (1991), Fletcher et al. (1996), Laguette et al. (2011)
Superantigen (Sag)	Singly spliced	MMTV/regulatory/accessory	Type II transmembrane protein essential for efficient transmission of milk-borne MMTV from the gut to the mammary gland via lymphocytes.	Golovkina et al. (1992), Korman et al. (1992), Held et al. (1993)
Regulator of export of MMTV mRNA (Rem)	Doubly spliced	MMTV/regulatory	Nuclear export of unspliced RNA by binding to Rem-responsive element (RmRE).	Indik et al. (2005), Mertz et al. (2005)
dUTPase or DU	Singly, double, or multiply spliced, or unspliced only	Betaretroviruses and nonprimate lentiviruses/regulatory	Contributes to virulence, viral mutation rate, and replication in nondividing cells.	Elder et al. (1992), Bergman et al. (1994), Payne and Elder (2001), Hizi and Herzig (2015)

## Retroviral virion

Retroviruses are spherical enveloped particles with an average diameter ranging between 80 and 120 nm, containing two copies of the gRNA (Fig. 2B; Coffin 1997). Each copy of the retroviral gRNA is a single-stranded, linear, nonsegmented, positive-sense RNA molecule, ranging from 7 to 12 kb in length (Coffin 1997). These gRNA strands are like cellular genes, featuring a cap at the 5' end and a polyadenylated tail (polyA tail) at the 3' end. In a literal sense, the retroviral genome should be considered as a “pseudodiploid” viral genome since the two copies of the gRNA lead to only one provirus in cells infected with one retrovirus particle (King et al. 2008). The two copies of the single-stranded gRNAs in retroviral particles are present as a complex consisting of two molecules, forming a 70S dimer through hydrogen bonds by the dimer linkage structure (extended-duplex) present at the 5′ end of the viral gRNA (Fig. 2B) (Canaani et al. 1973, Mangel et al. 1974, Kung et al. 1976, Bender et al. 1978, Murti et al. 1981, Paillart et al. 1996b, Greatorex 2004). This arrangement sets retroviral gRNA apart from cellular or subgenomic viral RNAs and may be responsible for its specific recognition into the virus particle during the RNA packaging/encapsidation process.

## Retroviral genome organization

**Figure 3.**
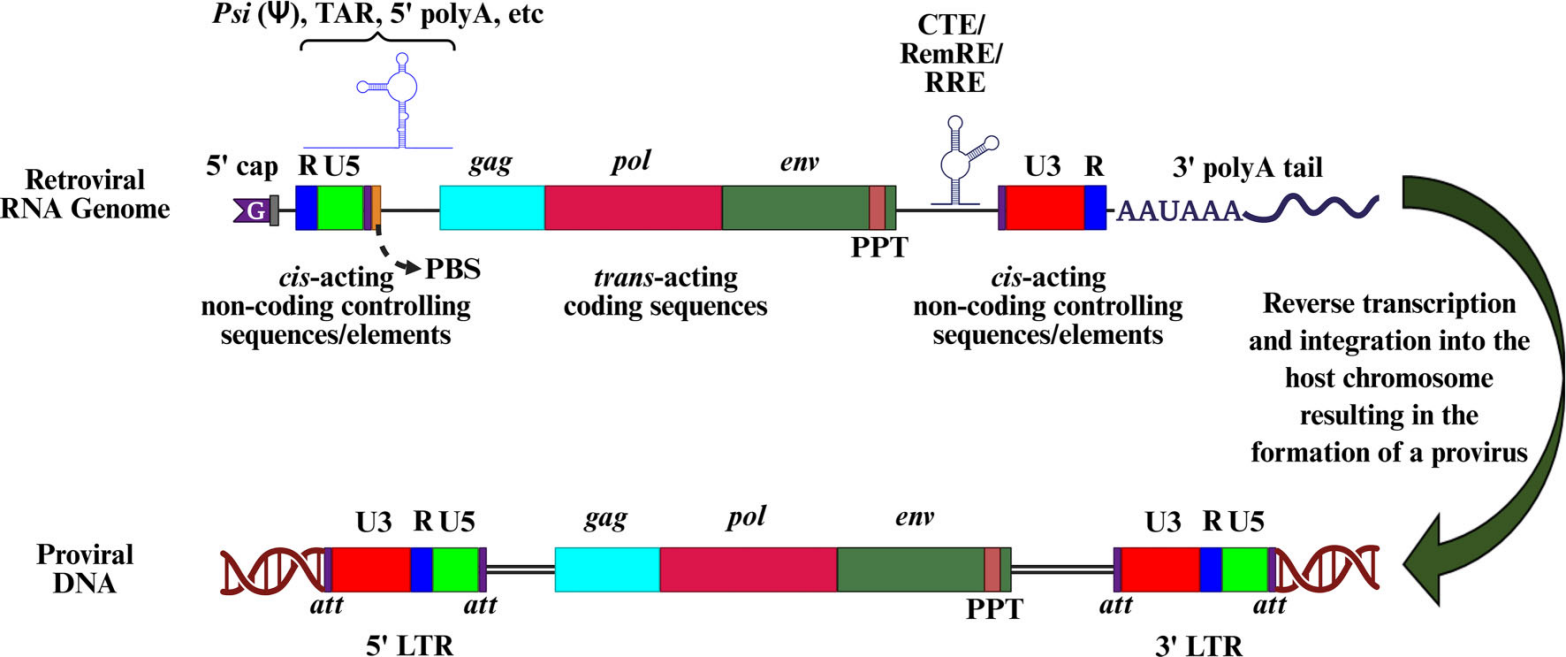
Comparison of the retroviral genomic RNA with its proviral DNA integrated into the host genome. Retroviral genome shown as an RNA monomer that is used during reverse transcription to generate a fully double stranded DNA molecule. The reverse transcribed DNA is then integrated into the host chromosomes to form proviral DNA. The figure highlights important *cis*- and *trans*-acting features observed in the retroviral genome and needed for reverse transcription, nuclear export, and RNA packaging. *Psi* (Ψ), Packaging sequences or signals; PBS, primer binding site; PPT, polypurine tract; U5 and U3, sequences unique to the 5′ and 3′ ends of the RNA genome; R, Repeat region; CTE, constitutive transport element; *att*, attachment sites used during integration; TAR, the *trans*-activation response element; and RRE, the Rev response element. Figure made in BioRender.com.

As illustrated in Fig. 3, the RNA genome of retroviruses can be broadly classified into *cis*- and *trans*-acting sequences/elements. The *cis*-acting elements comprise of noncoding regulatory sequences, whereas the *trans*-acting elements are sequences coding for the structural and functional/enzymatic proteins of the virus (Fig. 3) (Coffin 1997). The *cis*-acting elements are generally confined to the 5′ and 3′ ends of the viral RNA genome, while the *trans*-acting elements occupy the center of the retroviral/proviral genome.

### Cis-acting noncoding sequences and/or elements

#### Repeat (R) region

The R region is named for its duplicated occurrence within the gRNA: one immediately after the 5′ end cap site and the second at the 3′ end just upstream of the posttranscriptionally added polyA tail (Fig. 3). Depending on the type of retrovirus, the length of the R region can be between 15 and 250 nucleotides (nts). For example, in MMTV, R is only 15 nts long, whereas in *lentiviruses*, R is roughly 100 nts in length, and in HTLV-1, R is ∼250 nts long (Sodroski et al. 1984, Moore et al. 1987, Coffin 1997, Ohi and Clever 2000). The R region is extremely important since transcription is initiated at the start of the 5′ R region (often referred to as + 1R) and continues until the end of the 3′ R region. The R region also has a fundamental role in reverse transcription. Additionally, in some retroviruses, R encodes RNA signals that are critical for transcriptional regulation. For instance, in *lentiviruses*, the R region forms a structured element known as the *trans*-activation response element (TAR), which is essential for recruiting the viral transcriptional *trans*-activator protein Tat, indispensable for the transcription and elongation of gRNA transcripts (Laspia et al. 1989, Keen et al. 1997).

#### Sequences unique to 5′ and 3′ ends

The “unique” sequence/region, located immediately after the R but before the beginning of the primer binding site (PBS) at the 5′ end, is termed “U5” (a region unique to the 5′ end; Fig. 3). Similarly, the region located immediately upstream of R at the 3′ end is termed “U3” (a region unique to the 3′ end; Coffin 1997, Hunter 2008). These U5 and U3 regions also contain the attachment (*att*) sites required for the integration of the reverse-transcribed RNA into the host genome (see the U5 and U3 *att* sites in Fig. 3; Roth et al. 1989, Kulkosky and Skalka 1994, Coffin 1997, Masuda et al. 1998). During reverse transcription of retroviral RNA, the U5 and U3 regions are duplicated and relocated to the 3′ and 5′ ends of the genome, forming the complete long terminal repeats (LTRs; Fig. 3). The U3 region also encompasses transcriptional control elements required for transcript initiation from the provirus (Cullen et al. 1985, Graves et al. 1985, Coffin 1997, van Opijnen et al. 2004, Karn and Stoltzfus 2012). This includes the core promoter and multiple enhancer sequences that respond to both cellular and, in some cases, viral transcriptional activator proteins (Jones et al. 1986, Nabel and Baltimore 1987, Garcia et al. 1989, Coffin 1997, Pereira et al. 2000). In some retroviruses, such as MMTV, the U3 region of the 5′ LTR also harbors NREs and hormone-responsive elements. The specific characteristics of the enhancer sequences play a crucial role in determining the tissue specificity of viral replication and, consequently, the pathogenesis of the virus (Coffin 1997, Reed-Inderbitzin and Maury 2003, Hunter 2008). The U3 region occasionally includes coding sequences for viral proteins as well. For example, the U3 region of the 3′ LTR of HIV-1 partially harbors sequences of the *nef* gene (Adachi et al. 1986), while the U3 region of 3′ LTR of MMTV encompasses the entire *sag* open reading frame (ORF) and the second exon of *rem* gene (Korman et al. 1992).

#### PBS

The PBS is a highly conserved primary sequence motif in all retroviruses without any exception. It consists of ∼18 nts positioned immediately downstream of the U5 region (Fig. 3). The PBS is complementary to the 3′ end of specific cellular tRNAs, which interestingly serve as the primer for the initiation of reverse transcription by providing a free “3′ OH” group (Marquet et al. 1995, Coffin 1997, Hunter 2008, Isel et al. 2010). Different retroviruses use different host tRNAs as primers. For example, *lentiviruses* use Lys^−1, 2, 3^, whereas MMTV and MPMV utilize Lys^−3^ and Lys^−1, 2^, respectively (Coffin 1997). In addition to its pivotal role in the initiation of reverse transcription, a recent study reported the involvement of PBS in the packaging of MMTV gRNA (Chameettachal et al. 2021).

#### Polypurine tract

Similar to the PBS, all retroviruses contain a polypurine tract (PPT) near the 3′ end of the viral genome, upstream of the U3 region (Fig. 3). The PPT consists of fewer than 20 ribonucleotides, including stretches of adenines and guanines (Huber and Richardson 1990, Coffin 1997, Rausch and Le Grice 2004). During reverse transcription, after the negative strand DNA is synthesized, the PPT being resistant to RNase H cleavage stays intact in the RNA–DNA hybrid, while the RNase H continues to hydrolyze the RNA, allowing PPT to serve as a primer for the initiation of plus strand DNA synthesis (Coffin 1997).

### Transacting coding sequences and/or regions

**Figure 4.**
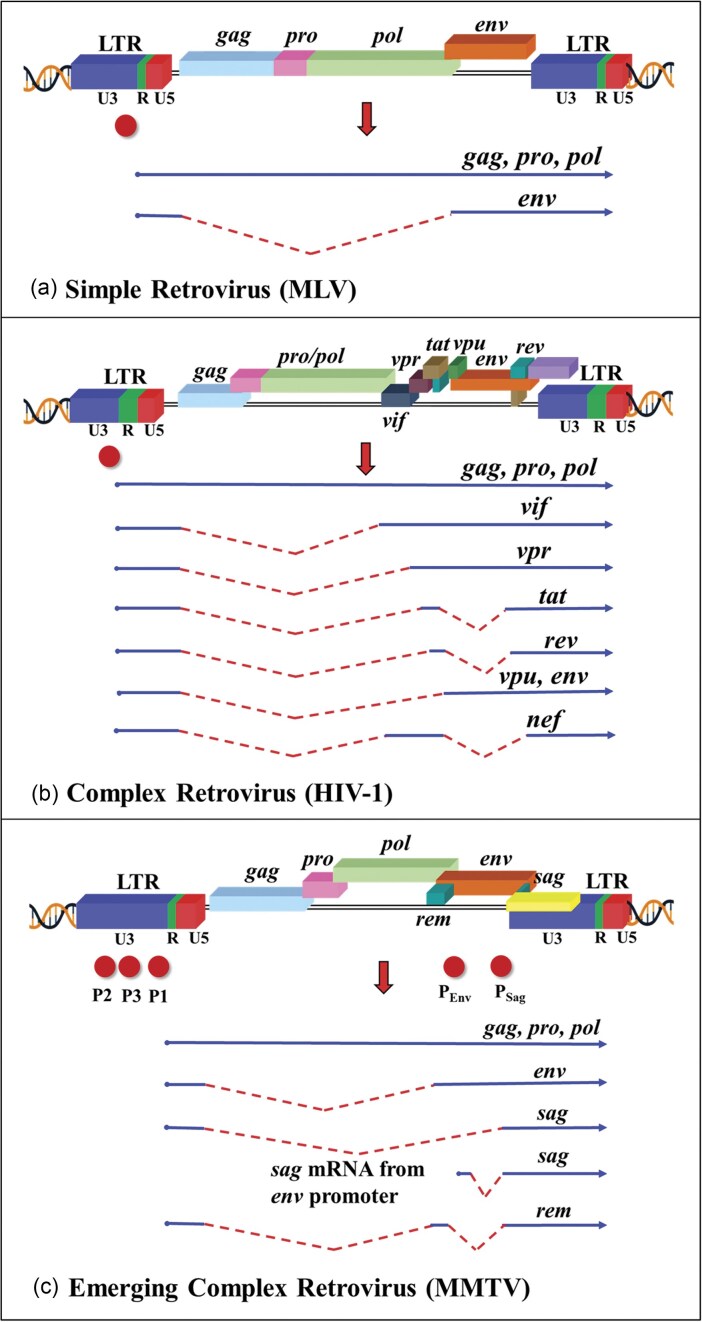
Illustration of simple and complex retroviral genomes, along with the splicing patterns of their full-length unspliced RNA genome in: (a) murine leukemia virus (MLV), a simple retrovirus, (b) human immunodeficiency virus type 1 (HIV-1), a prototypic complex retrovirus, and (c) MMTV, now recognized as an emerging complex retrovirus. The red circle marks the location of the retroviral promoters. See text for details pertaining to each step. Figure made in BioRender.com.

The protein-coding region of a retrovirus, often referred to as the *trans*-acting sequences/regions, is flanked by *cis*-acting sequences/regions at the 5′ and 3′ ends (Fig. 3). All replication-competent retroviruses contain four canonical ORFs: *gag, protease* (*Pro or PR*), *Pol*, and *Env*. These genes are essential for synthesizing the structural (Gag), enzymatic (Pro or PR and Pol), and envelope (Env) proteins. Retroviruses that express only these four canonical genes are commonly referred to as simple retroviruses (Fig. 4). Simple retroviruses (such as ALV, MLV, MPMV, and mammalian type C retroviruses) lack the ability to encode any accessory or regulatory proteins and express only one spliced RNA transcript for the expression of the Env protein (Coffin 1997, Hunter 2008) (Fig. 4). In contrast, complex retroviruses, such as *lentiviruses*, MMTV, and HTLV-1, harbor sequences necessary for coding regulatory and/or accessory proteins that are expressed from doubly or multiply spliced mRNAs that are crucial for the replication and survival of the virus in the host cells (Coffin 1997, Hunter 2008) (Fig. 4; Table 1).

#### Gag

Without exception, Gag is expressed from the first ORF within the unspliced gRNA as a precursor polyprotein in cells infected by retroviruses (Freed and Martin 2013, Goff 2013). Despite their unique features, all retroviral Gag proteins share three essential domains, arranged from N- to C-terminus as follows: matrix (MA), capsid (CA), and nucleocapsid (NC). MA plays a crucial role in directing Gag or the viral core to the plasma membrane during virus assembly. CA forms the fundamental structure of the viral core, while NC is responsible for directly coating the gRNA. The role of Gag is indispensable for the packaging of gRNA into the forming virus particle (Freed 1998, Bell and Lever 2013).

#### Protease (Pro or PR) and Pol

PR is responsible for proteolytic cleavages during virion maturation to make mature Gag and Pol proteins. Similar to Gag, the PR and Pol are encoded as part of a polyprotein. This polyprotein includes PR, RT, RNase H, and IN, and is cleaved by the viral PR itself to produce the functional enzymes (Coffin 1997, Hunter 2008). Depending on the retrovirus type, PR is encoded either as part of Gag (a Gag–Pro fusion protein; e.g. RSV and ASLV), as part of Pol (as a Pro–Pol fusion protein; e.g. HIV-1 and MLV), or as a separate ORF from Gag (as Pro; e.g. MPMV and MMTV; Fig. 4).

Some retroviruses like MMTV contain an additional gene called dut, which encodes a deoxyuridine triphosphatase (dUTPase or DU). In retroviruses that express dUTPase, the dut gene is present at two distinct positions within the genome, potentially offering different evolutionary pathways for these viruses. In *betaretroviruses* (such as MMTV and MPMV), the dUTPase is encoded by both the *Gag* and *Pro* ORFs and is a proteolytically cleavage product of the Gag–Pro polyprotein (Bergman et al. 1994, Barabás et al. 2003, Hizi and Herzig 2015). Conversely, in nonprimate *lentiviruses* [such as FIV, equine infectious anemia virus (EIAV), maedi-visna virus (MVV), and caprine encephalitis virus (CAEV)], the gene encoding dUTPase is found within the *Pol* ORF, making it a product of proteolytic cleavage of the Pol polyprotein precursor (Elder et al. 1992). It has been suggested that dUTPase plays roles in viral virulence, viral mutation, and facilitate productive viral replication in nondividing cells by providing nucleotide precursors (Payne and Elder 2001, Hizi and Herzig 2015).

#### Env glycoprotein

Enveloped viruses, including retroviruses, face a unique challenge (and perhaps an advantage) during cell entry due to their protective lipid membrane derived from the host cell membrane. Most retroviruses overcome this obstacle by fusing with the host cell membrane during infection using their Env protein, which interacts with a receptor on the host cell surface (Coffin 1997, Hunter 2008). Without exception, the Env protein of retroviruses is synthesized from a singly spliced mRNA as a precursor protein, which is then cleaved by cellular proteases into the external glycoprotein gp120 (SU), which noncovalently associates with the gp41 (TM) domain to form the gp120–gp41 heterotrimer (McCune et al. 1988, Willey et al. 1988, Freed and Martin 1995). The gp120 and gp41 proteins are presented on the cell surface as an envelope complex and are incorporated into viral envelope during the budding process of viral particles from the infected cells (White et al. 2010). Upon infection, gp120 mediated the interaction of HIV-1 with its receptor, CD4 (cluster of differentiation 4) cell molecule and the coreceptor, which could vary depending on the cell types, while gp41 facilitates the fusion of viral and cellular lipid membranes (Kwong et al. 1998, Eckert and Kim 2001, Melikyan 2011). Proper incorporation of envelope proteins into the viral particle is essential for ability of the virus to recognize and enter host cells (Fig. 4).

#### Accessory and/or regulatory genes in complex retroviruses

In addition to the canonical Gag, Pro, Pol, and Env proteins found in all retroviruses, complex retroviruses also contain genes encoding accessory and/or regulatory proteins. These proteins play crucial roles in viral replication *in vivo* and are often involved in regulating viral gene expression, replication, pathogenesis, and disease progression. The genes encoding these accessory/regulatory proteins are typically located downstream of the *pol* and *env* genes (Fig. 4). Table 1 provides a comprehensive review with regards to the expression of these accessory/regulatory proteins and the significant roles they play in the life cycle of a specific retroviral group. As can be seen, they serve disparate roles from regulating transcription, nuclear export of unspliced mRNAs, virion release, and nuclear import of preintegration complex (PIC), to facilitating virus infectivity and spread in the host, affecting viral virulence, apoptosis, host immune responses, persistence of infected cells, cell cycle, DNA damage response, and so on. (Table 1).

## Retroviral life cycle

The retroviruses life cycle consists of a series of highly interconnected and intricate steps that exploit both viral and host factors to facilitate their successful replication. Despite the presence of 11 distinct genera (Fig. 1), the overall steps of virus replication are highly similar among these groups, revealing the fundamental processes used by retroviruses to ensure their survival (Coffin 1997). The retroviral life cycle can be divided into an “early” phase” that primarily utilizes viral factors packaged within the infectious virion to initiate and execute each step of the viral life cycle with the help of host proteins. This phase consists of infection (entry) of the virus particle into the host cell, reverse transcription to create a DNA copy from the RNA genome, and integration of the viral DNA genome (now a provirus) into the host chromosome. This is followed by a “late phase,” consisting of transcription, translation, virus assembly, and egress from the infected cells. Both phases exploit important host factors and cellular pathways for proper execution and completion of the virus life cycle, as described in detail in the following sections.

### Attachment, membrane fusion, and entry

**Figure 5.**
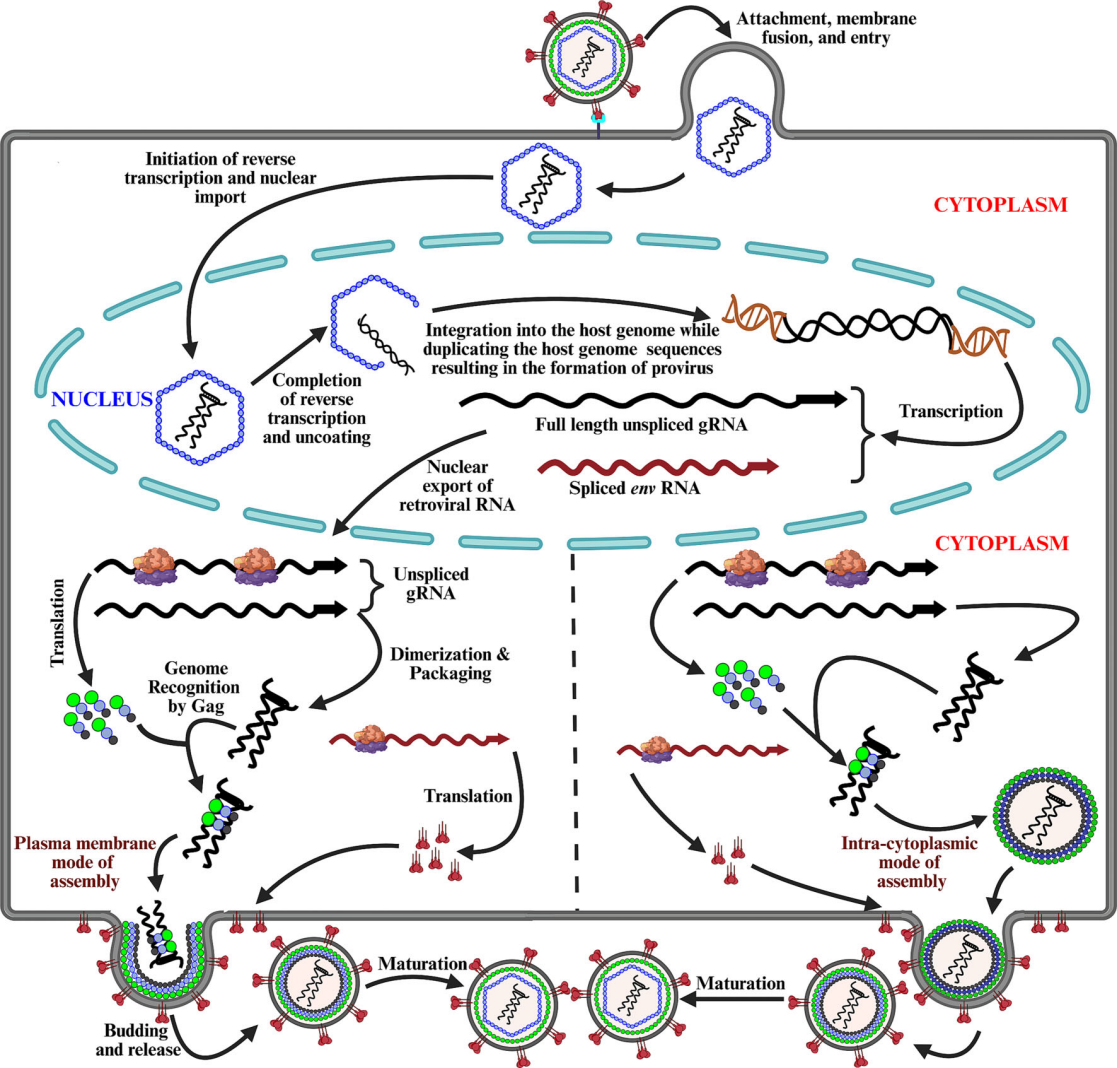
Illustration of a typical retroviral life cycle showing the various stages of retroviral replication. The figure highlights the two modes of assembly observed in different retroviruses, which can occur either directly at the plasma membrane, or in the cytoplasm followed by transport to the plasma membrane where budding virus particles acquire the envelope glycoproteins. See text for details pertaining to each step. Figure made in BioRender.com.

The first step in the retroviral life cycle is attachment, which involves interactions between the cell receptor and the SU subunit of the Env glycoprotein (Fig. 5). This interaction induces a significant structural modification in the Env protein that acts as a trimer, enabling the penetration of the TM (gp41) domain into the target cell membrane, ultimately causing fusion between the viral and cellular lipid bilayers (White et al. 2008, Swanstrom et al. 2017). For example, during HIV-1 infection, the viral SU domain (gp120) binds to CD4 receptors expressed on the cells of the immune system, namely T lymphocytes (T-helper cells), cells of the monocyte lineage (microglia and macrophages), and dendritic cells, inducing a conformational change in both CD4 and gp120. This change allows gp120 to bind to the coreceptors, either CXCR4 or CCR5 present on the cells of the immune system, depending upon the cell type (Bleul et al. 1997, Zaitseva et al. 1997, Berger et al. 1999). Coreceptor binding triggers further conformational changes and dissociation of the trimer of gp120–gp41 heterotrimer. This dissociation helps gp41 penetrate the plasma membrane, facilitating fusion of the viral envelope with the plasma membrane, allowing the deposition of the CA containing the viral genetic material into the cytoplasm of the host cell (Carlson et al. 2008, Blumenthal et al. 2012).

The mechanism facilitating conformational changes during retroviral infection has been intensely studied over the years. Unlike avian influenza viruses, where the conformational change is triggered by low pH (Skehel et al. 1982), most retroviruses use pH-independent pathways for fusion and entry into the host cell (Stein et al. 1987, McClure et al. 1988, 1990). However, ALV and MMTV are exceptions, as their entry mechanisms require a low pH step following receptor binding (Mothes et al. 2000, Ross 2000).

Since the discovery that homozygous defects in the HIV-1 coreceptor (CCR5) in individuals can prevent HIV-1 infection despite multiple exposures, many retroviral receptors, coreceptors, and cofactors have been identified (Liu et al. 1996, Overbaugh et al. 2001). Several retroviruses can exploit the same cell surface molecule as their receptor, indicating the range of host species they can infect. For example, several *gammaretroviruses* [cat endogenous RD-114 virus, spleen necrosis virus (SNV), and reticuloendotheliosis virus A (Rev-A)], *betaretroviruses* [simian retroviruses (SRVs) and MPMV)], and human endogenous retrovirus W (HERV-W) use a common receptor called Receptor for D-type retrovirus, often referred to as the RD-114, which is ubiquitously expressed in various human cells (Sommerfelt et al. 1990, Kewalramani et al. 1992, Koo et al. 1992, Tailor et al. 1999b, Blond et al. 2000). Similarly, the XPR1 protein expressed in *Xenopus laevis* oocytes is a common receptor for both xenotropic and polytropic MLVs (Battini et al. 1999, Tailor et al. 1999a). In the case of MMTV, it exploits the mouse transferrin 1 protein as its cellular receptor (Ross 2000). Intriguingly, an immunoreceptor tyrosine activation motif within the MMTV Env has been implicated in transforming mammary epithelial cells, suggesting that MMTV Env not only facilitates viral infection, but may also play a role in mammary carcinogenesis (Ross et al. 2006). Despite considerable progress in identifying receptors for most retroviruses, the mechanism of entry still needs to be deciphered molecularly for many for a better understanding of how retroviruses use cellular proteins for entry into permissive cells.

### Reverse transcription, nuclear import, and uncoating

**Figure 6.**
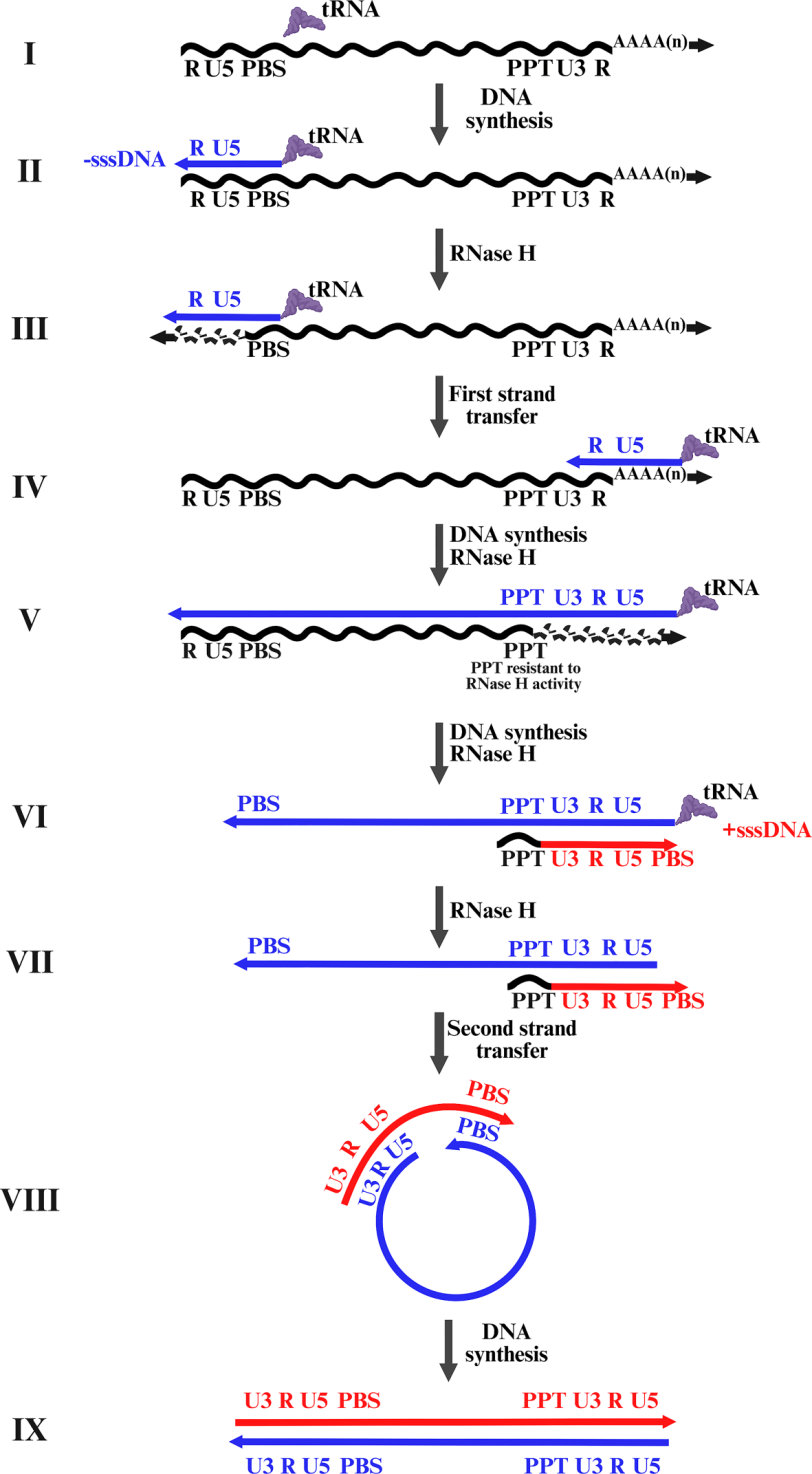
Schematic representation of the steps involved in retroviral reverse transcription, beginning with the annealing of a specific host tRNA (depending on the retrovirus) to the PBS on the viral gRNA. −sssDNA and +sssDNA, minus- and plus-strand strong-stop DNA, RT; reverse transcriptase; and PPT, polypurine tract. See text for details pertaining to each step. Figure made in BioRender.com.

Reverse transcription among retroviruses is a key step in their life cycle during which the viral RNA genome is converted into double-stranded DNA (dsDNA) by the enzyme RT (Baltimore 1970, Temin and Mizutani 1970). Besides retroviruses, RT is also employed during the replication of hepadnaviruses and retrotransposons (Bavand et al. 1989, Boeke 2003, Hughes 2015, Rausch et al. 2017). The various steps involved in retroviral reverse transcription are illustrated in Fig. 6, and include: (i) initiation of minus strand DNA synthesis using tRNA as a primer, (ii) synthesis of minus strand strong stop DNA, (iii) removal of the RNA part of the RNA–DNA hybrid by RNase H activity, (iv) the first template switching or negative DNA strand transfer using the R regions at the two flanks, (v) completion of minus strand DNA synthesis and degradation of the RNA genome by RNase H from the DNA–RNA hybrid, (vi) PPT’s resistance to RNase H degradation and its role as a primer for positive DNA strand synthesis, (vii) the second template switching or plus DNA strand transfer and removal of the tRNA primer, and (viii and ix) completion of minus- as well as plus-strand DNA synthesis, resulting in dsDNA with two complete flanking LTRs. These steps (as shown in Fig. 6) have been extensively documented, with some variations across different retroviral systems (Gilboa et al. 1979, Kung et al. 1981, Lee and Coffin 1991, Julias et al. 2002, Boyer et al. 2004, Hu and Hughes 2012, Hughes 2015).

While it is known that reverse transcription initiates inside the CA once it is deposited in the cytoplasm upon virus fusion, there has been a longstanding debate about where and when it is completed in different retroviruses. It was previously believed that after reverse transcription, the viral core uncoats in the cytoplasm, and only the PIC enters the nucleus (Dvorin and Malim 2003, Bukrinsky 2004, Suzuki and Craigie 2007). However, using HIV-1 as a model, it has now been established that reverse transcription begins in the cytoplasm and is completed within the intact CA in the nucleus (Fig. 5). Recent studies have shown that in HIV-1, the intact CA containing the reverse transcription complex, which includes dimerized RNA, RT, CA, NC, IN, Vpr, and host factors such as APOBEC3G gains access to the nucleus (Fig. 5) (Burdick et al. 2020, Dharan et al. 2020, Selyutina et al. 2020, Li et al. 2021, Müller et al. 2021).

The critical role of the CA in HIV-1 replication has been definitively established using a cell-free system (Christensen et al. 2020). While it is understandable that during HIV-1 replication, nuclear pore entry of the intact CA is facilitated by its bullet (conical) shape/architecture, it remains largely unclear how other retroviruses containing a spheroid or polyhedral intact CA pass through the nuclear pore to gain entry into the nucleus, since that would pose a formidable challenge (Mattei et al. 2016, Perilla and Gronenborn 2016, Qu et al. 2018, Zila et al. 2021). In addition, recent studies have shown that HIV-1 CA can enter the nucleus of nondividing cells with the help of host factors like the cleavage and polyadenylation specificity factor 6 (CPSF6) and nuclear pore complex (NPC) proteins, NUP153 and NUP358 (Matreyek et al. 2013, Buffone et al. 2018, Bejarano et al. 2019, Burdick et al. 2020, Dharan et al. 2020, Selyutina et al. 2020, Zila et al. 2021). However, other retroviruses that lack these interactions with NPC proteins must wait for nuclear membrane breakdown during mitosis to gain entry into the nucleus (Harel et al. 1981, Roe et al. 1993). Therefore, it remains largely unclear where reverse transcription takes place for most of the retroviruses. A recent study using different cell lines has shown that, similar to HIV-1, MLV initiates reverse transcription in the cytoplasm and completes it within the nucleus in both dividing and nondividing cells. During this process, MLV interacts with NPC proteins NUP358 and NUP62 to enter the nucleus in nondividing cells (Salas-Briceno et al. 2024). These findings are of great significance because they challenge the central dogma that a simple retrovirus’s intact CA cannot gain access to the nucleus in nondividing cells. Future studies will likely enhance our understanding of reverse transcription and other steps in the life cycle of simple retroviruses.

During reverse transcription, retroviruses acquire mutations which are primarily due to the poor fidelity of RT. However, cellular RNA polymerase II, along with various viral and host factors, also influences retroviral mutations, particularly in HIV-1 (O’Neil et al. 2002, Mangeat et al. 2003, Chiu and Greene 2006, Hughes 2015). The low fidelity of RT is primarily because retroviral RTs lack proofreading mechanisms (such as 3′ exonuclease activity), making them error-prone, especially for actively replicating retroviruses such as HIV-1 compared to others, such as HTLV and BLV (Battula and Loeb 1976, Starcich et al. 1986, Preston et al. 1988, Ratner et al. 1991, Dube et al. 1993, Willems et al. 1993). Based on cell-free assays designed to read mutations during DNA-dependent DNA synthesis, it has been shown that AMV and MLV RTs introduce nearly 10-fold fewer mutations when compared to HIV-1 RT (Roberts et al. 1988, 1989, Barrioluengo et al. 2012). Interestingly, assays designed to read mutations during RNA-dependent DNA synthesis reveal no significant difference in mutation rates among AMV, MLV, and HIV-1 RTs, indicating that HIV-1 RT’s misincorporation rate is higher during DNA-dependent DNA synthesis (Roberts et al. 1988, Boyer et al. 1992, Ji and Loeb 1992, Yu and Goodman 1992, Bakhanashvili and Hizi 1993, Kerr and Anderson 1997). Furthermore, using a single round of replication assay, Mansky and Temin (1995) elegantly demonstrated that the mutation rate of HIV-1 is much lower than that reported for purified HIV-1 RT using cell-free assays, suggesting that the accuracy of purified HIV-1 RT might not truly represent the extent of genetic variation occurring during natural infection.

Besides RT’s low fidelity as a cause of mutations, the process of reverse transcription itself allows genetic variability resulting from recombination during the first template switch or negative DNA strand transfer. Considerable work has been carried out using different retroviral systems to establish whether template switching occurs in an ordered fashion or not, especially given that retroviral particles harbor two copies of the gRNA, but no consensus has yet been developed. For example, it was initially thought that the first strand transfer during reverse transcription was exclusively intermolecular (interstrand), and the second strand transfer was intramolecular. This meant that reverse transcription required both gRNAs for making a single linear reverse-transcribed DNA molecule (Panganiban and Fiore 1988). A few years later, another study reported that the first strand transfer can be either intramolecular or intermolecular, while the second strand transfer is always intramolecular (Hu and Temin 1990). It has also been reported that both the first and the second strand transfers are intramolecular, indicating that one RNA template is sufficient for the completion of DNA synthesis (Jones et al. 1994). To further add to this quandary, in the case of HIV-1, van Wamel and Berkhout (1998) demonstrated that the first strand transfer is a random process and both intramolecular and intermolecular transfers make nearly equal contributions during this process. Irrespective of the nature of the first strand transfer to be intramolecular, intermolecular, or random, they all are likely to contribute toward genetic variation among retroviruses during DNA synthesis, owing to the numerous errors occurring during reverse transcription (Temin 1993).

Uncoating, being an essential step in the retrovirus life cycle, has been intensely investigated with regards to its timing and exact location. It was previously believed that for almost all retroviruses, after reverse transcription, uncoating of the viral core happened in the cytoplasm, and only the PIC containing reverse-transcribed viral DNA, along with host and viral proteins, entered into the nucleus. In contrast to this, HIV-1 PICs have been found to contain CA molecules; furthermore, some recent studies report the presence of intact CA and CA-like structures inside the nucleus, challenging the prevailing notions about retroviral CA uncoating (Burdick et al. 2020, Dharan et al. 2020, Selyutina et al. 2020, Müller et al. 2021). Consistent with these findings, even in the case of a simple retrovirus such as MLV, it has recently been shown that the viral CA can gain access to the nucleus (Salas-Briceno et al. 2024), prompting further scrutiny of CA uncoating. Thus, for at least HIV-1, once the CA is inside the nucleus, reverse transcription and PIC formation are completed, and uncoating occurs near the integration site ~1.5 hours before integration due to the mounting physical pressure exerted by the newly reverse transcribed DNA (Fig. 5) (Rankovic et al. 2017, Burdick et al. 2020, Dharan et al. 2020, Li et al. 2021, Müller et al. 2021, Yu et al. 2022).

### Integration

Integration is a crucial step in the retrovirus life cycle, during which the viral cDNA, synthesized through reverse transcription, is permanently inserted into the host cell genome (Fig. 5). This process ensures stable maintenance and replication of the viral genome within the host. Integration enables the viral DNA to be transcribed and translated using the host’s cellular machinery, facilitating persistent infection and contributing to the pathogenesis of retroviral diseases. Soon after the completion of reverse transcription, the newly reverse transcribed dsDNA within the PIC (also known as the stable synaptic complex or intasome) is intertwined with the IN enzyme, which mediates the integration process. The integrated retroviral DNA is now called a “provirus” (Fig. 5). The concept of a provirus was put forward by Howard Temin in 1964, which at that time was a very provocative idea (Temin 1964). It took over a decade for this thought to be scientifically accepted, followed by a series of studies that revealed the molecular characteristics of the integrated provirus amongst many retroviruses. The importance of integration in the retroviral life cycle can be appreciated from the fact that mutations that specifically hinder integration impede replication of different retroviruses. The direct evidence for the process of integration and involvement of the retroviral IN enzyme in this process was derived by employing *in vitro* systems. These studies demonstrated that PIC-containing reverse-transcribed dsDNA could be integrated into a heterologous DNA (Fujiwara and Mizuuchi 1988, Fujiwara and Craigie 1989, Bushman et al. 1990, Craigie et al. 1990, Katz et al. 1990, Lee and Coffin 1990, Engelman et al. 1991, Brown 1997).

**Figure 7.**
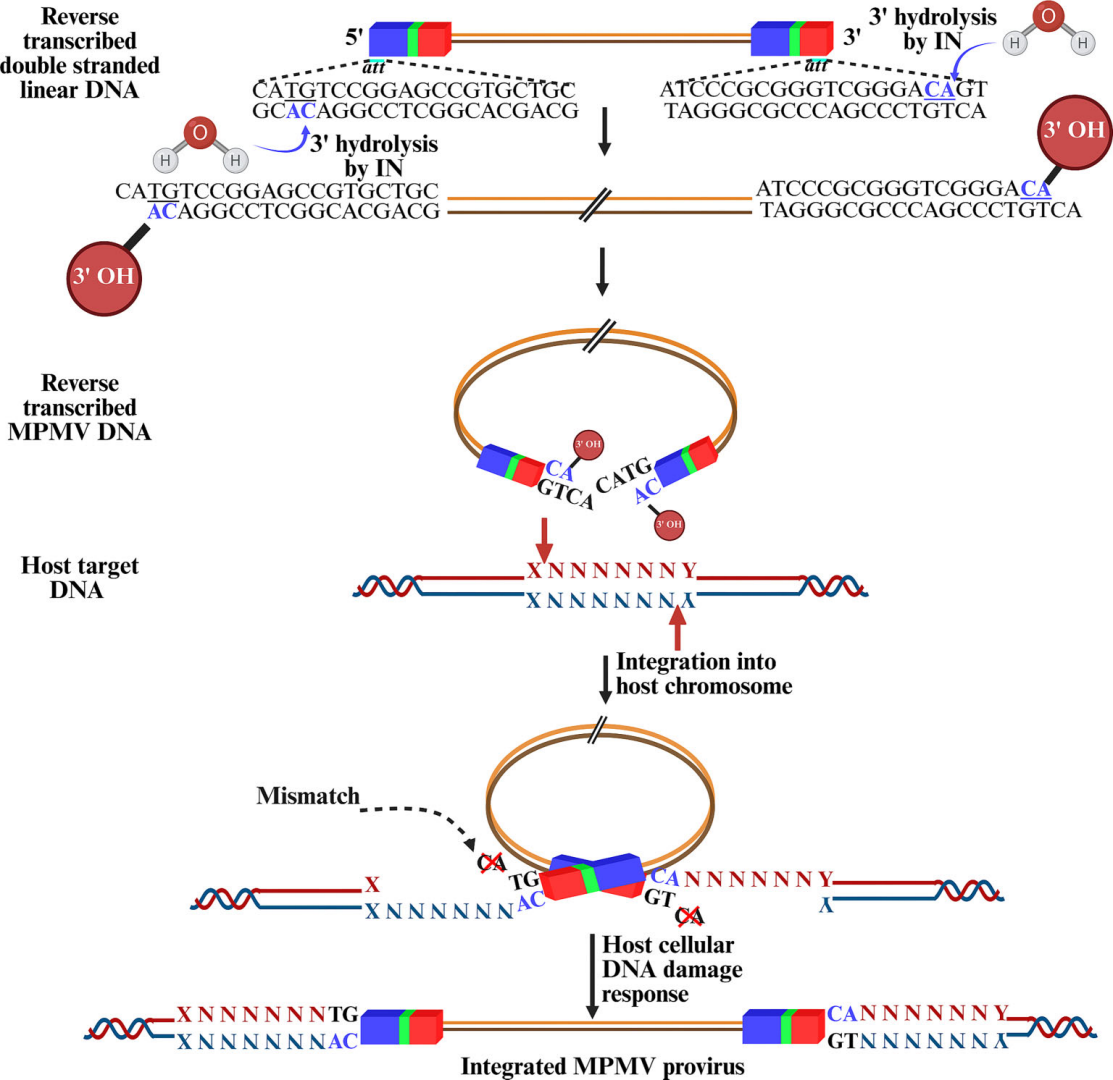
Illustration of the sequential steps involved in retroviral integration, using MPMV as an example. N, any of the four nucleotides in the host DNA; X and Y, host nucleotides that are six host base pairs away from the inserted viral DNA with 5′ TG and 3′ CA ends. See text for details pertaining to each step. Figure made in BioRender.com.

Although most of our current understanding about retroviral integration is derived from the early work conducted in MLV, later studies conducted in HIV-1 have significantly enhanced our understanding of this process (Bowerman et al. 1989, Brown et al. 1989, Craigie and Bushman 2012, Lusic and Siliciano 2017). The IN enzyme, which is from the Pol ORF, is expressed as a polyprotein along with Gag–Pol or Gag–Pro–Pol (depending upon the retrovirus type). During virus particle maturation, this polyprotein is cleaved by the viral PR enzyme into RT, RNase H, and IN components (Donehower and Varmus 1984, Panganiban and Temin 1984, Schwartzberg et al. 1984). Briefly, the process of integration starts as PIC or intasome (nucleoprotein complex containing IN tetramer) by positioning itself at the ends of the reverse-transcribed DNA (Fig. 7). Such a positioning allows interaction with the target capture complex within the host’s chromosomal DNA, leading to strand transfer and permanently connecting the viral and cellular DNA molecules (Maertens et al. 2010, Hare et al. 2012).

Depending upon the retrovirus type, IN acts on ~16–20 terminal nucleotides of the LTR (Colicelli and Goff 1988, Withers-Ward et al. 1994, Masuda et al. 1998, Brown et al. 1999). Characteristically, the process of integration of the reverse transcribed DNA in retroviruses involves two basic components: (i) the IN enzyme discussed above, and (ii) *cis*-acting sequences known as the U3 and U5 attachment (*att*) sites at the flanking ends of the reverse transcribed double stranded linear DNA (Fig. 3; Table 2). These *cis*-acting *att* sites are present in the form of imperfect inverted repeats and are both necessary and sufficient for integration both *in vitro* and *in vivo* (Fig. 7) (Colicelli and Goff 1988, Katzman et al. 1989, Bushman et al. 1990, Murphy et al. 1993, Brown et al. 1999). The *att* sites contain terminal, phylogenetically conserved CA and TG dinucleotides in the U5 and U3 ends of the blunt-ended DNA and play an important role during retroviral integration (Fig. 7; Table 2). However, the sequences internal to these dinucleotides are not well conserved, even though they are needed for ideal IN activity during integration (Bushman and Craigie 1991, Brown 1997, Desfarges and Ciuffi 2010, Ali et al. 2016). Interestingly, the requirement for the canonical CA dinucleotide at the end of U3 does not seem to be very rigorous, as shown in the case of RSV, where the CA dinucleotide is not required for integration of the U3 end of viral DNA (Oh et al. 2008). It has also been proposed that the RSV IN may recognize the U3 and U5 ends differently, with the sequence requirements at the U5 end being much more stringent than at the U3 end (Oh et al. 2008).

**Table 2.** Integration site selection of retroviruses.

**Retrovirus types**	**Preferred site for integration**	**Duplication flanking the integrated provirus**	**U3 and U5 *att* sites** Phylogenetically conserved TG/CA dinucleotides are shown in red and underlined.
*Alpharetroviruses* (ALV)	Weak preference for actively transcribing regions and no preference for transcription start regions (Mitchell et al. 2004, Narezkina et al. 2004)	6 nts (Hishinuma et al. 1981, Hughes et al. 1981)	**U3** ***att**:* 5′ AATGTAGTCTTATGC 3′ 3′ TTACATCAGAATACG 5′ **U5 *att***: 5′ AGCAGAAGGCTTCATT 3′ 3′ TCGTCTTCCGAAGTAA 5′ (Katzman et al. 1989)
*Betaretroviruses* (MMTV)	Random integration pattern (Faschinger et al. 2008, Konstantoulas and Indik 2014)	6 nts (Majors and Varmus 1981)	**U3 *att***: 5′ AATGCCGCGCCTGCAGCAGA 3′ 3′ TTACGGCGCGGACGTCGTCT 5′ **U5 *att***: 5′GGTCGGCCGACTGCGGCAGC 3′ 3′CCAGCCGGCTGACGCCGTCG 5′
*Gammaretroviruses* (MLV)	Strong preference for the actively transcribing regions, especially the 5′ end of the transcribing region (Wu et al. 2003, Mitchell et al. 2004).	4 nts (Dhar et al. 1980, Shoemaker et al. 1980)	**U3 *att***: 5′ AATGAAAGACCCC 3′ 3′ TTACTTTCTGGGG 5′ **U5 *att***: 5′ GGGGTCTTTCATT 3′ 3′ CCCCAGAAAGTAA 5′ (Brown et al. 1989)
*Deltaretroviurses* (HTLV-1)	Weak preference for the transcribing regions (Leclercq et al. 2000, Derse et al. 2007).	6 nts (Chou et al. 1996)	**U3 *att***: 5′ TGACAATGACCATGAGCCCC 3′ 3′ ACTGTTACTGGTACTCGGGG 5′ **U5 *att***: 5′ AGAGAAATTTAGTACACAGT 3′ 3′ TCTCTTTAAATCATGTGTCA 5′ (Ratner et al. 1991, Balakrishnan and Jonsson 1997)
*Epsilonretroviruses* (WDSV)	Unknown	Unknown	Unknown
*Lentiviruses* (HIV-1)	Strong preference for the entire actively transcribing region(s) (Schröder et al. 2002, Mitchell et al. 2004, Schaller et al. 2011).	5 nts (Vincent et al. 1990, Vink et al. 1990)	**U3 *att***: 5′ ACTGGAAGGGCTAATTCACT 3′ 3′ TGACCTTCCCGATTAAGTGA 5′ **U5 *att***: 5′ TGTGGAAAATCTCTAGCAGT 3′ 3′ ACACCTTTTAGAGATCGTCA 5′ (Masuda et al. 1998)
*Simiispumaviruses* (HFV)	Modest preference for transcription initiation sites but do not integrate preferentially within the genes (Nowrouzi et al. 2006, Trobridge et al. 2006).	4 nts (Schweizer et al. 1993, Neves et al. 1998)	**U3 *att***: 5′ GGTGTGGTGGAATG 3′ 3′ CCACACCACCTTAC 5′ **U5 *att***: 5′ AAATTCCATGACAA 3′ 3′ TTTAAGGTACTGTT 5′ (Neves et al. 1998)

During the process of viral DNA integration into the host cell genome, a series of well-synchronized steps must take place, as outlined briefly and illustrated in Fig. 7.

- First and foremost, the flanking termini of the newly reverse-transcribed viral DNA must be processed. During this 3′ end processing, IN removes the few nucleotides (di- or trinucleotides, depending upon the retrovirus) by hydrolyzing the phosphodiester bond at both the U3 and U5 att sites downstream of the phylogenetically conserved CA dinucleotide, generating staggered ends (Fujiwara and Mizuuchi 1988, Brown et al. 1989, Roth et al. 1989). Such an arrangement leads to the attachment of the 3′-hydroxyl group to the 5′-CA-3′ dinucleotides.
- Secondly, the intasome containing the processed viral DNA attaches to the host cell DNA, which facilitates the cleavage of the host cell DNA in a staggered fashion by IN using viral DNA 3′-hydroxyls as nucleophiles (Fujiwara and Mizuuchi 1988, Bushman and Craigie 1991, Engelman et al. 1991).
- Next, during a transesterification reaction, the processed CA-3′ end of viral DNA joins to the opposing strands of host cell DNA, while the unprocessed viral DNA 5′ ends do not join during this process (Engelman et al. 1991). During the repair of the single-stranded gaps, primarily cellular enzymes, and to a certain extent RT and IN, are responsible for removing the two unpaired nucleotides from the 5′ ends of the viral DNA and repairing the single-stranded gaps (Acel et al. 1998, Yoder and Bushman 2000).
- The resultant provirus now contains a short duplication of the target DNA sequence flanking the integrated provirus and terminates with the dinucleotides 5′-TG and CA-3′. Interestingly, these same 5′-TG and CA-3′ dinucleotides are also found at the termini of certain eukaryotic and prokaryotic transposable elements, as well as in all retrotransposons (Lee and Harshey 2003). The size of the duplicated target sequences (between 4 and 6 nts) depends on the type of retrovirus. For example, a duplication of 4 nts is observed for MLV, 6 for HTLV-1, ALV, and MMTV, and 5 for HIV-1 and other lentiviruses (Table 2).

Given the lack of sequence conservation of *att* sites among retroviruses, the process of proviral integration into the host cell DNA might be expected to be random. However, it is now becoming clearer that certain retroviruses like HIV tend to target gene-rich, transcriptionally active, euchromatic regions within the host’s chromosome to integrate. This is because integrating at a specific site could either enhance or repress transcription of the integrated proviral DNA. For example, proviral integration in the transcriptionally active regions could potentially favor expression of the viral genes, whereas integration in the transcriptionally silent regions (heterochromatin) may not be in favor of viral expression and may even force the virus to stay transcriptionally silent (Jordan et al. 2001, Lewinski et al. 2006, Einkauf et al. 2022, Teixeira et al. 2024). Equally consequential is the integration of the proviral DNA for the host, since it can either disrupt or enhance the expression of the host genes, resulting in altered and sometimes even fatal outcomes (Hacein-Bey-Abina et al. 2003, Bushman 2007, Montini et al. 2009, Desfarges and Ciuffi 2012).

Undoubtedly, the IN enzyme, which catalyses the integration process, is the crucial component of the PIC. However, over the years, it has been established that several other viral and host factors are also present in the PIC of some retroviruses. One of the leading factors is the lens epithelium-derived growth factor (LEDGF/p75), which has been shown to be an authentic cofactor for HIV-1 integration. LEDGF/p75 interacts with HIV-1 IN, influencing its integration activity, both *in vitro* and *in vivo* (Cherepanov et al. 2003, Ciuffi et al. 2005, Engelman and Cherepanov 2008, Craigie and Bushman 2014, Schemelev et al. 2024). However, while LEDGF/p75 is critical, it is not strictly essential for HIV-1 integration and replication. This assertion is supported by the fact that HIV-1 was able to replicate, *albeit* at a much-reduced level, in LEDGF knockdown cells, and the replication could be restored upon expression of LEDGF/p75 (Llano et al. 2006, Shun et al. 2007). Several plausible working models for the function of LEDGF/p75 during HIV-1 integration have been put forward: It (i) protects IN from proteasomal degradation, (ii) functions as a molecular adaptor for tethering the intasome to the target DNA, and (iii) augments strand transfer during integration (Emiliani et al. 2005, Llano et al. 2006, Shun et al. 2007). Contrary to this, LEDGF/p75 does not interact with INs from other retroviruses and has no binding ability to non-*lentiviruses* such as HTLV-2, MLV, and RSV (Llano et al. 2004, Busschots et al. 2005). However, MLV IN binds to the bromodomain and extraterminal (BET) domain family of proteins, especially the ET domain of Brd4, promoting MLV integration at transcriptional start sites (De Rijck et al. 2013, Larue et al. 2014, Crowe et al. 2016, Xing et al. 2021). Another important host factor identified in retroviral integration is CPSF6 that facilitates targeting of the HIV-1 PICs to transcriptionally active regions of the genome found deep within the center of the nucleus (Rasheedi et al. 2016, Achuthan et al. 2018, Singh et al. 2022a).

Additionally, other viral factors have been implicated in targeting retroviral integration. For example, it has been suggested that Gag-derived protein(s), especially CA, plays an important role during retroviral integration. Evidence toward this has been drawn from experiments that have shown that mutations in HIV-1 CA and its interactions with cyclophilin do not inhibit viral integration but favor integration into the genomic regions that are not transcriptionally active, as opposed to wild-type virus typically integrating into the transcriptionally active regions (Lewinski et al. 2006, Schaller et al. 2011, Engelman 2021). Further evidence shows that CA facilitates HIV-1 integration into transcriptionally active, gene-rich regions via interacting with CPSF that in fact helps connect the PIC to intranuclear trafficking pathways to facilitate entry into the inner regions of the nucleus (Chin et al. 2015, Achuthan et al. 2018).

Interestingly, in the case of HIV-1, IN has also been shown to have nonenzymatic/noncatalytic roles, affecting different steps in the viral life cycle beyond integration. Consistent with this, substitution mutations in the N-terminal domain of HIV-1 IN can nearly abolish proviral DNA formation and significantly reduce viral DNA synthesis, indicating that these mutations impact earlier steps in the viral life cycle, before integration (Masuda et al. 1995). Further studies have revealed that mutations in IN can affect uncoating, reverse transcription, nuclear import of viral DNA, and protein processing during viral assembly (Engelman et al. 1995, Masuda et al. 1995, Nakamura et al. 1997, Wu et al. 1999, Tsurutani et al. 2000, Ikeda et al. 2004, Briones et al. 2010). Furthermore, recently, it has also been shown that IN mutants, which are defective in binding to viral RNA, can cause altered HIV-1 particle morphology, with viral RNA located outside the core in mature, noninfectious virus particles (Kessl et al. 2016, Elliott and Kutluay 2020, Elliott et al. 2020). Finally, these studies also suggest that IN does not seem to have a role in RNA packaging since the amount of RNA in the IN-mutant virus particles was not reduced.

It has been observed that PIC or the intasome have greater inclination to integrate into bent target DNA that is wrapped with histones or transcription factors in comparison to the naked DNA (Müller and Varmus 1994, Pruss et al. 1994, Maskell et al. 2015, Matysiak et al. 2017). Furthermore, some studies have also suggested that palindromic (pal) motifs in the target DNA might influence IN activity during site selection in a virus-specific manner. Briefly, there seems to be a consensus, especially noticed in the retroviral integration site alignments, that a weak pal motif is often present in the target DNA sequences that are recognized by IN (Stevens and Griffith 1996, Holman and Coffin 2005, Wu et al. 2005, Miklík et al. 2023). Thus, various retroviruses have adapted to favor different integration sites depending upon the chromatin state (Desfarges and Ciuffi 2010). Based on several studies conducted over the years, it is now possible to develop a consensus on where each retrovirus genus prefers to integrate its reverse transcribed DNA. Table 2 summarizes the preferred integration site(s) for different retroviruses along with the pertinent references. For instance, *gammaretroviruses* like MLV and GaLV prefer integrating near transcriptional start sites and CpG islands, which are found within promoters and enhancer sequences. Similarly, *lentiviruses* like HIV-1 also prefer transcriptionally active regions, but integrate within the intragenic region, not at their 5′ ends. The *simiispumaviruses* are more similar to the *gammaretroviruses* with some preference for transcriptionally start sites instead of insertion within genes. On the other hand, *alpharetroviruses* like RSV show a less selective and more random integration profile with weak preference for transcriptionally active sites. The *deltaretroviruses* like HTLV-1, are similar to the *alphaviruses* with only a weak preference for transcriptionally active regions. Compared to these viruses, the *betaretroviruses* like MMTV has shown the most random pattern of integration amongst all retroviruses, while not much is known about the integration pattern of *epsilonretroviruses*.

The differences in integration site preferences thus suggest that both viral (the IN enzyme specificity) and host factors [such as the chromatin state (open more favored than closed) and presence of host factors like LEDGF, CPSF6, BET, and others] influence the pattern of integration shown by each retroviral genus.

### Transcription

Transcription from the integrated proviral DNA is a crucial step in the retrovirus life cycle, facilitating the production of viral RNAs and proteins necessary for the generation of new virus particles. During this process, the integrated proviral DNA serves as a template for transcription to the host’s cellular machinery (Fig. 5). Transcription of the proviral DNA is initiated in the U3 region of the 5′ LTR, which contains the promoter and other regulatory sequences. The promoter sequences within the U3 region are recognized by the host’s RNA polymerase II, along with various transcription factors that bind to these sequences to initiate transcription. Transcription from the proviral DNA results in a primary RNA transcript that must remain unspliced to be packaged as gRNA into the newly assembling virions, ensuring the continuity of the viral life cycle. This unspliced transcript also translates to produce Gag, Pro, and Pol polyproteins. Additionally, the unspliced transcript must be singly spliced in all retroviruses, without exception, to generate the *envelope* (*env*) mRNA. In complex retroviruses, the unspliced transcript undergoes multiple splicing events, generating various mRNA transcripts that are translated into accessory/regulatory proteins (Fig. 4).

As shown in Fig. 4, the U3 region of the 5′ end LTR acts as the nerve center, controlling transcription from the integrated provirus. All retroviruses, except for MMTV and foamy viruses, have only one promoter in the LTR. In the case of MMTV, multiple promoters have been described, with three reported within U3, while two reported within *env*. The first U3 promoter (P1) is the classical promoter near the U3-R border, while the other two promoters are found ∼650 and 415 nts upstream of P1, respectively (Rouault et al. 2007). All three U3 promoters have the potential to transcribe the known MMTV genes, including *sag*, The major MMTV promoter within the *env* gene is constitutively active, unlike the major U3 promoter that is hormonally regulated. It is located downstream of about one-third of the *env* start site and can only transcribe the *sag* gene from a novel spliced mRNA (Fig. 4C) (Wheeler et al. 1983, Jarvis et al. 1994, Mustafa et al. 2000). The second promoter reported in *env* is located right before the start of the 3′ LTR with B cell specificity, and the ability to express only *sag* from an unspliced mRNA (Arroyo et al. 1997). The U3 region of MMTV also harbors several transcription control elements, such as a mammary gland and other enhancers, negative regulatory elements (NREs), and a hormone response element (HRE) that controls the activation of the U3 promoters (Mink et al. 1990). The HRE is found upstream of the transcription start site of P1, requiring steroid hormones for its induction and activation of transcription in a tissue-specific manner (Cato et al. 1987, Bansal and Latchman 1990, Günzburg and Salmons 1992). This is similar to the two promoters observed in foamy viruses. They contain the classical LTR promoter that expresses the structural genes Gag/Pro/Pol, but additionally also have an internal promoter (IP) located toward the 3′ end of the *env* gene that can express only the two early regulatory genes, *tas* and *bet*. The basal activity of both promoters can be *trans*-activated by the Tas (*trans*-activator of *spumavirus*) protein (Baunach et al. 1993, Winkler et al. 1997, Omoto et al. 2004). Similar to MMTV, the basal activity of the IP is stronger than the LTR promoter due to the presence of an NRE in the LTR promoter. Interestingly, Tas can activate both the LTR and IPs using *cis*-acting response elements, an activity that is tissue specific (Löchelt et al. 1993, Campbell et al. 1994, Mergia 1994, Wei et al. 2022).

**Figure 8.**
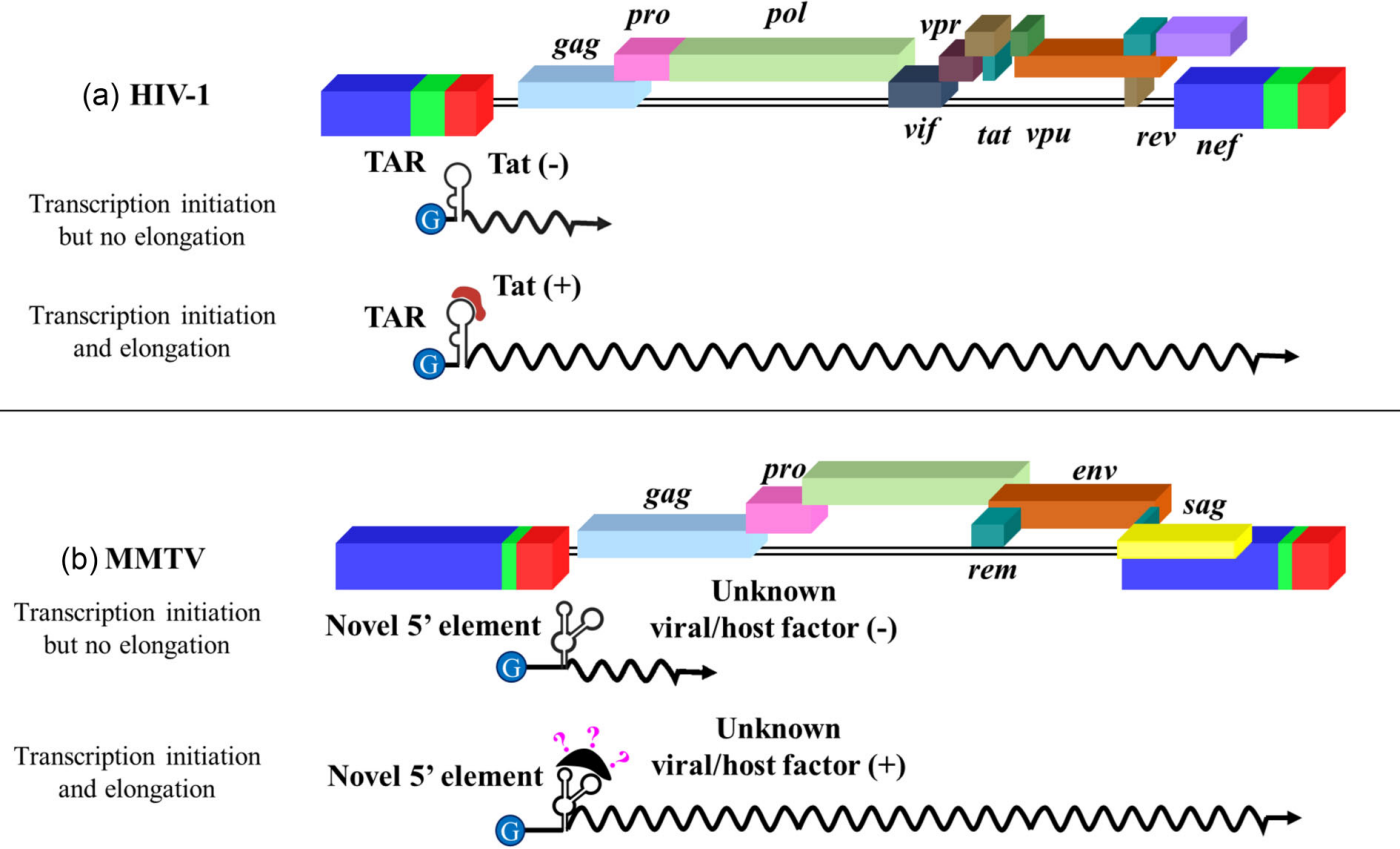
Illustration of the function of (a) Tat/TAR in the transcriptional regulation of human immunodeficiency virus type 1 (HIV-1), compared to the emerging *trans*-activation system recently identified in the (b) MMTV. See text for details. Figure made in BioRender.com.

Interestingly, retroviral LTR promoters function similarly to eukaryotic promoters. This is evident from the fact that retroviral promoters contain sequences that act as transcriptional enhancers and interact with viral or cellular *trans*-activating factors. For example, the HIV-1 promoter region includes a TAR element with a stem-loop structural motif in the R region (Fig. 8A). The potent viral *trans*-activator Tat interacts with this stem-loop motif, enhancing the transcription and elongation of viral transcripts (Berkhout et al. 1989, Keen et al. 1997, Barboric and Peterlin 2005, Das et al. 2011, Schulze-Gahmen and Hurley 2018). In the absence of TAR–Tat interaction, transcription can initiate, though inefficiently, and transcripts are not elongated, halting virus replication (Fig. 8A). Following the Tat–TAR interaction, the host super elongation complex, which includes the cellular positive transcription elongation factor p-TEFb and polymerase-associated factor 1 (PAF-1), is recruited to further boost transcription, significantly increasing the production of viral RNA transcripts (Fig. 8A) (Peterlin and Price 2006, Tahirov et al. 2010, Gu et al. 2014). It is now well-known that RNA polymerase II temporarily halts during transcript elongation, a pause stabilized by the negative elongation factor (NELF) and the 5,6-Dichloro-1-β-D-ribofuranosylbenzimidazole (DRB) sensitivity-inducing factor (DSIF) (Nechaev et al. 2010). To overcome this pause, RNA polymerase II, DSIF, and NELF must be phosphorylated by p-TEFb (Yu et al. 2015). During this process, the recruitment of the PAF complex via a flexible AFF4 scaffold ensures transcript elongation, providing the structural basis for the transition from pausing to active elongation (Feinberg et al. 1991, Chou et al. 2013, Vos et al. 2020).

Like HIV-1, HTLV-1 also contains *trans*-acting regulators of transcription in the form of Tax (protein) and Tax responsive element (TRE) in the LTR (Sodroski et al. 1984, Cann et al. 1985, Felber et al. 1985, Fujisawa et al. 1985). However, unlike HIV-1 Tat, Tax does not bind directly to TRE. Instead, it enhances transcription by facilitating the binding of the host transcription factor cAMP response element binding/activating transcription factor (CREB/ATF) to TRE (Franklin et al. 1993, Suzuki et al. 1993, Yin and Gaynor 1996). In *spumaviruses*, since transcription from the LTR promoter depends on Tas (*trans*-activator of *spumavirus*), which is expressed from the IP, use of the IP allows enough Tas to accumulate, facilitating its efficient binding to the LTR promoter rather than to the IP. This enables efficient transcription of viral transcripts that express structural and enzymatic proteins (Yang et al. 1997, Kang et al. 1998). In addition to Tas, many cellular factors are involved in regulating these two promoters, including AP1, BAG3, NF-ƙB, PKC, JNK, NF-AT, and so on. This has important biological consequences for the viral infection; while both promoters can be *trans*-activated by Tas during lytic infections, only the IP is activated by Tas during persistent infections, affecting the outcome of the infection for the cell (Meiering et al. 2001).

Recent studies on MMTV have identified a novel 12-nt-long *cis*-acting element located immediately downstream of the major splice donor, which is crucial for MMTV gRNA transcription, elongation, and stability (Fig. 8B) (Akhlaq et al. 2018). It has been proposed that, like HIV-1 TAR and HTLV-1 Tax, this MMTV novel 5′ end element may also contain viral and/or cellular protein-binding site(s) to modulate its function. Interestingly, this MMTV 5′ novel element responds to both HIV-1 Tat and HTLV-1 Tax and to the MMTV genome in a dose-dependent manner (Khader et al. 2024). Furthermore, *trans*-activation by both Tat and Tax involves pTEFb, which is intricately associated with both HIV-1 Tat/TAR and HTLV Tax/TRE *trans*-activation. These findings make MMTV the first *betaretrovirus* to potentially encode a Tat/TAR- and Tax/TRE-like transcription regulatory systems.

Retroviruses use the host transcriptional machinery to transcribe, cap [add a methyl guanosine (G) group at the 5′ end], and polyadenylate viral mRNAs at the 3′ end, including gRNA. In a eukaryotic cell, all these steps are obligatorily required for nuclear export of the RNAs and their subsequent translation (Jackson and Standart 1990, Eckner et al. 1991, Sachs 1993). Polyadenylation is a complex process involving several steps, during which the polyA signal must be recognized, the RNA cleaved at the polyA cleavage site, and the polyA tail added. Similar to cellular RNAs, two primary signals at the 3′ end of the mRNAs play a major role in polyadenylation in retroviruses. The first is a conserved hexamer (AAUAAA) polyA signal, located ∼15–23 nts before the cleavage site. The second is the downstream element, comprising of a poorly conserved GU- or U-rich region located ∼20–30 nts downstream of the polyA signal (Humphrey and Proudfoot 1988, Manley 1988, Swain and Coffin 1989, Wickens 1990, Proudfoot 1991, Guntaka 1993, Chen et al. 1995a). It is worth mentioning that in the case of MMTV, instead of the conserved hexamer AAUAAA, an alternative AGUAAA hexamer is located ∼20 nts upstream of the transcriptional start site, serving as the polyA signal (Klemenz et al. 1981, Coffin 1997). Interestingly, for retroviruses like HTLV-1, HTLV-2, and BLV, which have the polyA signal in the U3 region, the R region is relatively long (over 200 nts), positioning the polyA signal at an unusually long distance from the polyadenylation site (Temin 1981, Derse and Casey 1986). In these cases, it is hypothesized that the higher-order structure of the RNA brings the polyA signal closer to the polyadenylation site, facilitating the 3′ end processing of the transcript (Ahmed et al. 1991). Polyadenylation is further facilitated by the binding of the cellular cleavage and polyadenylation specificity factor (CPSF) to the conserved polyA signal.

The presence of a polyA signal at the R-U5 junction positions the polyA signal at both the 5′ and 3′ ends of the transcript. This results in the inhibition of transcription from the 5′ polyA signal (Fig. 3). For example, in retroviruses (such as SNV, MLV, MPMV, lentiviruses, and foamy viruses), the polyA signal is present in the R region (downstream of the transcription start site), appearing at both the 5′ and the 3′ ends of the transcripts (Fig. 3). Retroviruses with this arrangement of the polyA signal have evolved mechanisms to prevent premature polyadenylation from the first polyA signal present in the 5′ LTR. This requires its occlusion and/or repression at the 5′ end to obviate premature termination of the transcripts (Honigman et al. 1985, Guntaka 1993). On the other hand, in retroviruses, such as RSV, AMV, MMTV, JRSV, BLV, HTLV-1, and HTLV-2, the polyA signal is present only in the U3 region and thus is not part of the transcript at the 5′ end. Therefore, these viruses do not require mechanisms to bypass the 5′ end polyA signal.

One possible mechanism that explains why the 5′ polyA sequence does not result in polyadenylation or is suppressed has been explored experimentally by various groups (Iwasaki and Temin 1990, Weichs an der Glon et al. 1991, Cherrington and Ganem 1992, DeZazzo et al. 1992). These groups interrogated the spacing between the cap site and the polyA signal and observed that increasing this distance facilitates efficient 3′-end processing. This scenario is somewhat similar to what has been observed in the cases of hepatitis B virus and cauliflower mosaic virus (Russnak and Ganem 1990, Sanfaçon and Hohn 1990). Another possible mechanism involves the usage of specific sequences in the U3 region upstream of the conserved hexamer, since these sequences are absent at the 5′ end of the transcript. These sequences have been shown to boost polyadenylation by improving CPSF binding both *in vitro* as well as *in vivo* (Brown et al. 1991, Valsamakis et al. 1992, Ashe et al. 1995, Gilmartin et al. 1995, Graveley and Gilmartin 1996). Structure prediction and biochemical approaches suggest that in HIV-1, the polyA signal forms a stem-loop motif that could facilitate suppression of the polyA signal at the 5′ end of the transcript (Berkhout et al. 1995, Das et al. 1999, Klasens et al. 1999). Additionally, involvement of splicing machinery has also been shown to influence the occlusion of the 5′ end polyA signal in the case of HIV-1 (Furger et al. 2001). Furthermore, in both HIV-1 and foamy viruses, the primary factor in splice-site-mediated suppression is the binding of U1 small nuclear ribonucleoprotein (U1 snRNP), part of the spliceosome complex, to the major splice donor site (Ashe et al. 1995, 1997, 2000, Schrom et al. 2013). In the case of MLV, which also contains functional polyA signal at the 5′ end of the transcript, suppression of the 5′ end polyA signal is not dependent on the splicing machinery and primarily relies on the weak nature of the 5′ end polyA signal, thereby evading the untimely polyadenylation at the 5′ end (Furger et al. 2001).

### Nucleocytoplasmic export of retroviral RNAs

**Figure 9.**
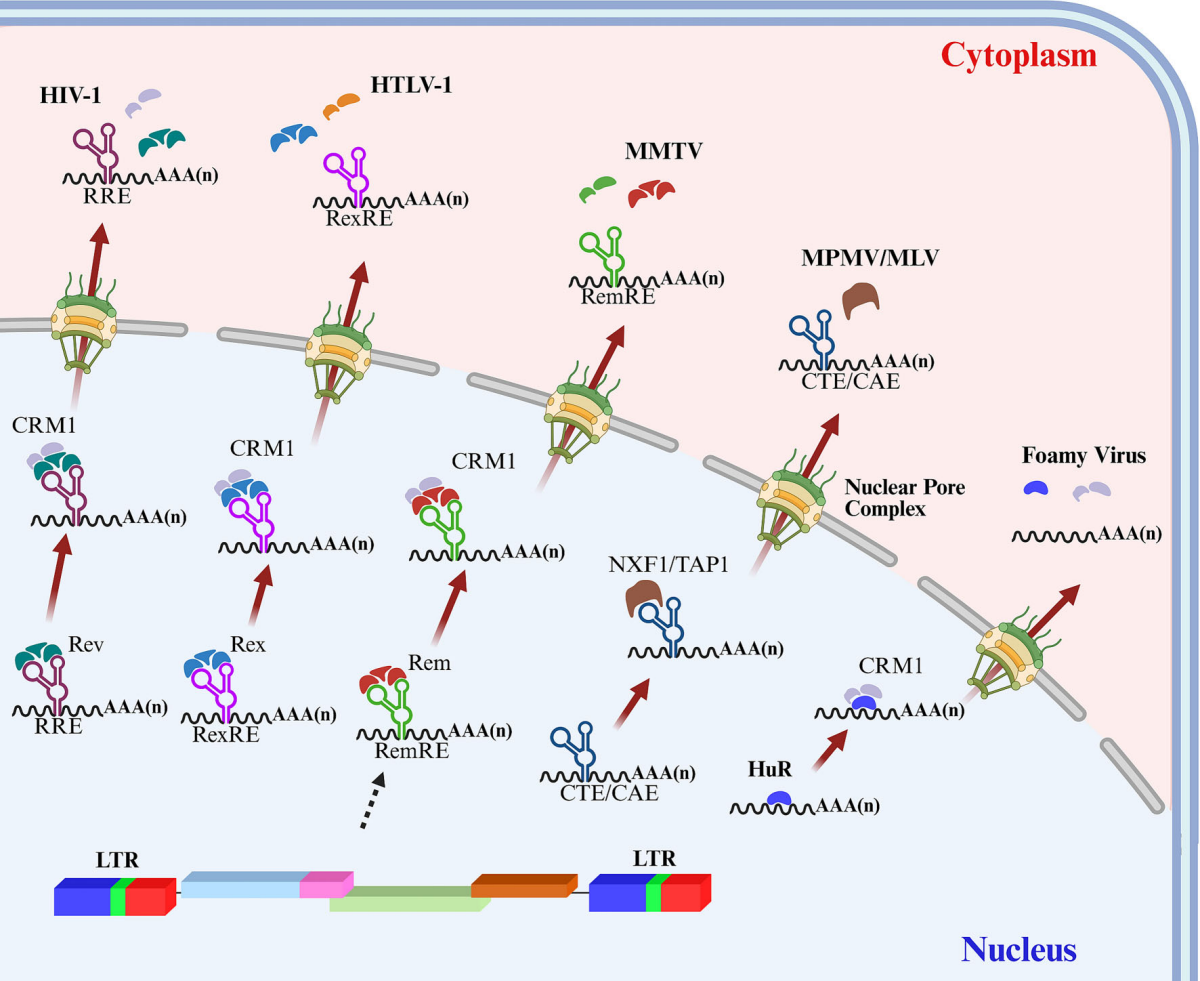
Illustration of the mechanisms used by different retroviruses for the nuclear export of their full-length unspliced RNA. HIV-1, human immunodeficiency virus type 1; HTLV-1, human T-lymphotropic virus type 1; MMTV, mouse mammary tumor virus; CRM1, chromosome region maintenance 1 protein; MPMV, Mason–Pfizer monkey virus; MLV, murine leukemia virus; CTE, constitutive transport element; CAE, cytoplasmic accumulation element; NXF1/TAP, nuclear RNA export factor 1; and HuR, human antigen R. See text for details. Figure made in BioRender.com.

Unlike cellular RNAs, which must undergo splicing to remove introns before being exported to the cytoplasm, retroviruses need to export both their unspliced and spliced RNAs from the nucleus to the cytoplasm. For retroviruses, the nuclear export of full-length unspliced RNA to the cytoplasm is vital because it serves two major functions in the retrovirus lifecycle: (i) it serves as the gRNA that can be packaged into assembling virions to continue the viral replication and (ii) it serves as a template for translating structural and enzymatic proteins important for virion formation. Therefore, retroviruses have evolved various mechanisms to subvert the cell’s splicing machinery, ensuring proper expression and export of their unspliced RNAs. Simple retroviruses primarily rely on *cis*-acting sequences to subvert the cell’s splicing machinery to downregulate splicing, including inefficient splice sites, negative regulatory sequences that inhibit splicing in *cis*, nonconsensus branchpoint sequences, and pyrimidine-rich tracts interspersed with purines to control the extent of splicing (Arrigo and Beemon 1988, Katz et al. 1988, Katz and Skalka 1990, Berberich and Stoltzfus 1991, Fu et al. 1991, McNally et al. 1991, McNally and Beemon 1992, Zhang and Stoltzfus 1995). Furthermore, they exploit the nuclear mRNA export machinery to bypass the splicing requirement for the safe export of their mRNAs. For instance, simple retroviruses like MPMV use *cis*-acting sequences known as constitutive transport element (CTE) which are positioned within the *env* gene overlapping with the 3′ LTR (Fig. 9) (Bray et al. 1994). Being present at the 3′ end, the CTE is common to all viral mRNAs, which interact with and recruit the cellular nuclear export factor 1 heterodimer (NXF1/NXT1, also known as TAP), for their export to the cytoplasm, just like cellular mRNAs (Fig. 9) (Ernst et al. 1997, Pasquinelli et al. 1997, Grüter et al. 1998, Bear et al. 1999). Thus, simple retroviruses require coordination between splicing and nuclear export pathways to ensure safe passage of their spliced and unspliced mRNAs to the cytoplasm for expression and virion assembly.

In contrast to simple retroviruses, complex retroviruses have evolved specialized mechanisms, involving both viral and cellular factors, for efficient export of unspliced RNA (Fig. 9). Complex retroviruses such as HIV-1 encode a 116 amino acid RNA-binding protein called the regulator of virion (Rev). The Rev protein binds to a conserved and highly structured *cis*-acting RNA motif, the Rev Responsive Element (RRE), found within the envelope region. Consequently, the RRE is present in both unspliced and singly spliced RNAs, facilitating their export from the nucleus (Hadzopoulou-Cladaras et al. 1989, Malim et al. 1989, 1990, Cochrane et al. 1990, Bai et al. 2014). To achieve this, the Rev/RRE complex recruits a host protein, the cellular chromosomal maintenance 1 (CRM1, also known as Exportin 1; XpoI) and Ran (RAs-related nuclear protein) also known as GTP-binding nuclear protein, to form the nuclear export complex that successfully exports these RNAs from nucleus to the cytoplasm (Fig. 9 and Table 1) (Neville et al. 1997, Askjaer et al. 1998, Bai et al. 2014, Booth et al. 2014, Schemelev et al. 2024). Without Rev, the unspliced and singly spliced RNAs build up in the nucleus and are eventually degraded (Malim et al. 1989).

Similar to HIV-1, the nuclear export of unspliced and singly spliced RNAs in HTLV-1 is controlled by the Rex response element (RexRE). The RexRE is a *cis*-acting RNA stem-loop structural motif located within the 3′ LTR and interacts with a virally encoded *trans*-acting protein called Rex (Fig. 9; Table 1) (Ahmed et al. 1990, Grassmann et al. 1991, Younis and Green 2005). Following the interaction between Rex and RexRE, RNAs are stabilized, splicing is inhibited, and they are subsequently exported out of the nucleus via a CRM1-dependent cellular pathway (Fig. 9) (Ahmed et al. 1990, Grassmann et al. 1991, Hakata et al. 1998, 2001, 2003, Nakano and Watanabe 2012).

Similarly, MMTV encodes a protein called Regulator of Export of MMTV mRNA (Rem), which interacts with the Rem Responsive Element (RmRE) present at the 3′end of the genome within the *env* gene, but overlapping the 3′ LTR. Like HIV-1, this element is found in both the unspliced and spliced RNAs (Fig. 9; Table 1) (Indik et al. 2005, Mertz et al. 2005, 2009, Müllner et al. 2008). The RmRE is predicted to form an RNA secondary structure with multiple stem loops, and it has been proposed that one of these stem loops serves as a docking site for the Rem to multimerize, mediating the nuclear export of unspliced RNA (Indik et al. 2005, Müllner et al. 2008, Mertz et al. 2009). Rem is homologous to the Rev/Rex/Rec/Rej proteins encoded by HIV, SIV, FIV, HTLV-1, HERV-K, and Jaagsiekte sheep retrovirus (JSRV), which also bind to their respective *cis*-acting RNA elements during RNA export (Hidaka et al. 1988, Cheng et al. 1990, Malim et al. 1990, Phillips et al. 1992, Löwer et al. 1995, Palmarini and Fan 2003). Intriguingly, MMTV, which was previously classified as a simple retrovirus, uses a mechanism similar to complex retroviruses for the transport of its unspliced RNA, but not the spliced Env mRNA. The Rem/RmRE complex also recruits CRM1 to form the nuclear export complex for exporting the unspliced RNA; however, MMTV Env expression is independent of CRM1 since it is independent of leptomycin B treatment, an antibiotic that inhibits CRM1-dependent mRNA export (Fig. 9; Table 1) (Müllner et al. 2008, Boeras et al. 2012, Hohenadl et al. 2012). This finding has prompted arguments for reclassifying MMTV as a complex retrovirus. Consistent with this assertion, MMTV RmRE can interact with HIV-1 Rev and HTLV-1 Rex to enhance expression in an MMTV-based reporter assay using human cells (Mertz et al. 2009).

Coming back to the transport elements of simple retroviruses, the MPMV CTE has been studied considerably and consists of a 234-nt long, structured RNA element that forms a stem loop (Fig. 9) (Bray et al. 1994, Rizvi et al. 1996a, Tabernero et al. 1996). CTE is essential for virus replication as it facilitates the nuclear export of unspliced RNA. Interestingly, CTE can also compensate for the function of the Rev/RRE and Rem/RmRE regulatory systems in *lentiviruses* and MMTV, respectively (Bray et al. 1994, Rizvi et al. 1996b, 2009). This is primarily because CTE does not bind to any viral factors, but as mentioned earlier, interacts with the cellular mRNA export factor TAP/NXF1 to efficiently export unspliced viral RNA (Fig. 9) (Tabernero et al. 1996, Ernst et al. 1997, Grüter et al. 1998, Bachi et al. 2000). Additionally, CTE has been shown to bind to RNA helicase A to fulfill its function (Tang et al. 1997, Reddy et al. 2000). A similar mechanism has also been observed in MLV, where a cytoplasmic accumulation element has been identified in the *pol* gene that interacts with NXF1 to export unspliced and spliced RNA transcripts from the nucleus to the cytoplasm (Fig. 9) (Pessel-Vivares et al. 2014, Sakuma et al. 2014). Similarly, RSV uses its direct repeats flanking the *src* gene for the nuclear export of its unspliced RNA in a CRM1-independent manner (Paca et al. 2000). Some evidence suggests that NFX1 and the DEAD-box RNA helicase (DBP5) may be involved in this process (LeBlanc et al. 2007).

In addition to the two retroviral nuclear export mechanisms discussed above, there exists a third mechanism employed by the foamy retroviruses. Interestingly, foamy viruses, do not express any virally encoded *trans*-acting proteins, and no known *cis*-acting RNA elements have been identified in them. Yet, they utilize a pathway that can be considered a blend of the other two mechanisms (Fig. 9). The prototype foamy virus achieves nuclear export of its unspliced RNA by interacting with human antigen R (HuR), a ubiquitously expressed nuclear RNA-binding shuttle protein known to be involved in the nuclear export of AU-rich elements-containing cellular mRNAs (Fig. 9) (Fan and Steitz 1998, Bodem et al. 2011). The RNA–HuR binding facilitates the recruitment of CRM1, successfully exporting the unspliced RNA from the nucleus to the cytoplasm.

This difference is not surprising since foamy viruses (as part of *Spumaretrovirinae* subfamily) display several other interesting exceptions to the typical features seen in the structure and replication of *Orthoretrovirinae*. In fact, they are considered the “most ancient” among retroviruses and display features in between those of *Orthoretrovirinae* and *Hepadnaviruses* (Lee et al. 2013). Some of the unique features of this subfamily include: (1) initiation of reverse transcription of the viral genome during the late stage of virus assembly, leading to the presence of fully or partially dsDNA in the released virions similar to that observed in *Hepadnaviruses*, (2) synthesis of Pro/Pol proteins from a separate spliced subgenomic mRNA, leading to their recruitment into virions via Gag C-terminal interactions rather than the classical Gag–Pro/Pol (or Gag/Pol) fusion, (3) an unusual requirement of Env for budding where Gag alone is not sufficient for release of virus particles, and (4) a unique Gag protein that lacks the classical domains observed in *Orthoretrovirinae*, such as the zinc finger motifs (cysteine–histidine boxes) and the plasma membrane-targeting signal. Absence of the membrane-targeting domain results in intracellular budding of foamy viruses into the endoplasmic/Golgi secretory pathway where they acquire Env, rather than the plasma membrane. Interestingly, this signal is found in one of the three glycine–arginine-rich rich sequences (GR motifs) observed in foamy virus Gag that compensate for the lack of zinc fingers important for encapsidation of genomic RNA (gRNA) in *Orthoretrovirinae* as well as Pol incorporation into the assembling virions (Calcraft et al. 2024, Lee et al. 2013, Müllers 2013, Lindemann and Rethwilm 2011, Linial 1999).

### Translation

**Figure 10.**
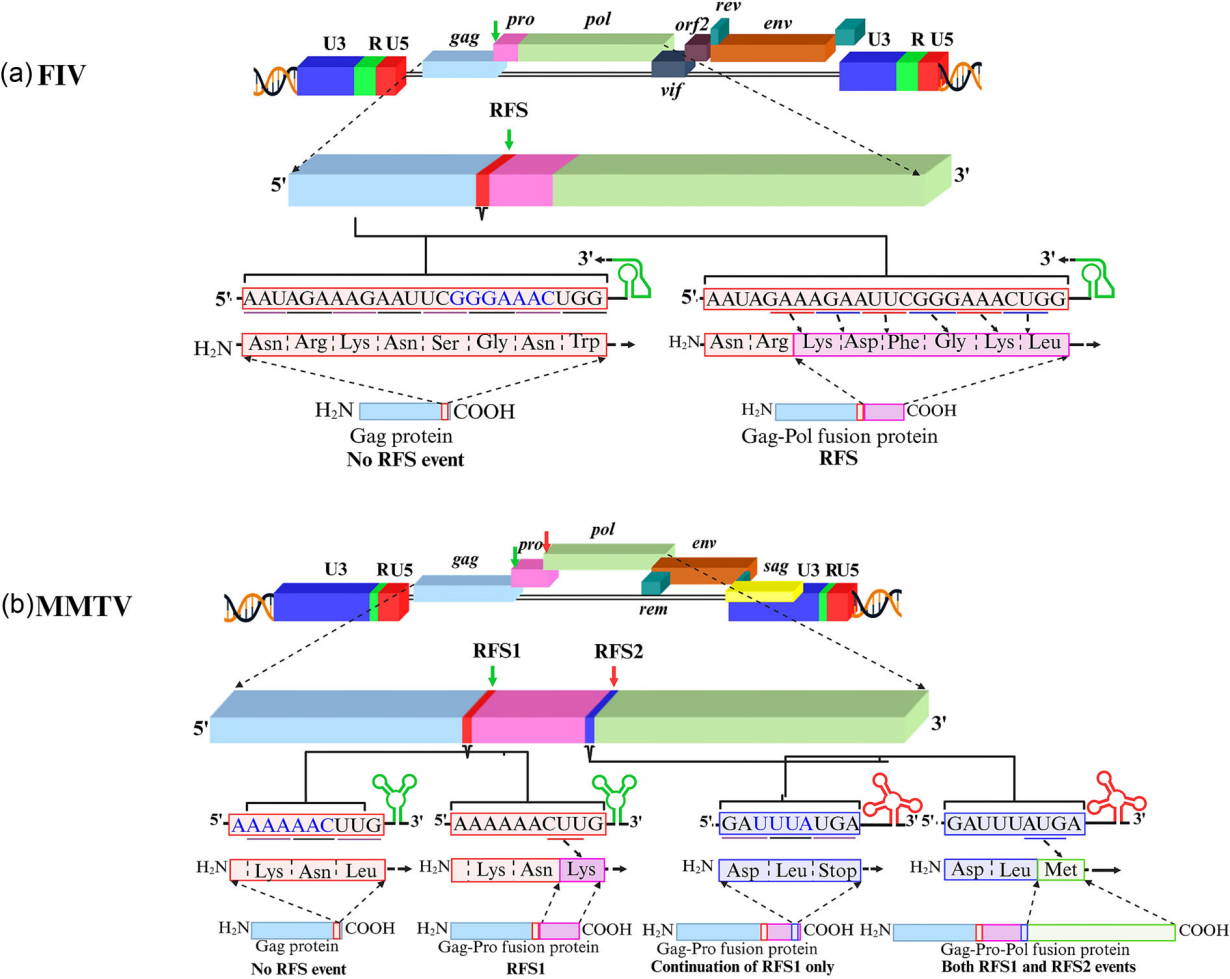
Illustration of the ribosomal frameshifting mechanisms employed by different retroviruses to translate overlapping genes from a single start codon (AUG). (a) In feline immunodeficiency virus (FIV) and other *lentiviruses*, ribosomes undergo a single −1 nt shift (indicated by the green downward arrow) just before the Gag stop codon, altering the reading frame from encoding the Gag precursor protein to the Gag–Pol polyprotein. (b) The MMTV utilizes a more complex mechanism involving two successive −1 frameshifts. The first shift (indicated by the first downward arrow) occurs before the Gag termination codon, switching the reading frame to produce the Gag–Pro polyprotein. A second −1 shift (indicated by the second downward arrow) occurs prior to the Pro stop codon, resulting in translation of the full Gag–Pro–Pol polyprotein. These frameshifting events require a reiterated sequence motif, known as ribosomal frameshift signal (RFS) and a downstream RNA secondary structure, typically a pseudoknot, located 5–8 nts after the frameshift site. See text for details. Figure made in BioRender.com.

In contrast to cellular RNA transcripts, which typically encode only one protein, retroviral unspliced RNA translates multiple proteins. This is primarily because different overlapping genes are encoded within the gRNA, such as the Gag–Pol, Gag–Pro, and Gag–Pro–Pol translational units (depending on the retrovirus type) that are expressed from the same start/initiation (AUG) codon (Fig. 4). In addition, most of them encode many other genes throughout the genome that are either singly or multiply-spliced with their own start and stop codons. To express the genes with the same AUG, retroviruses have evolved three main mechanisms: (1) splicing, (2) translational readthrough, and (3) ribosomal or translational frameshifting. Of the last two mechanisms mentioned, ribosomal frameshifting is more common in most retroviruses (Coffin 1997). The mechanism of translational readthrough involves suppressing the termination codon at the end of the *gag* gene, thereby maintaining the same reading frame during the translation of Gag and Pol, as has been observed in MLV (Yoshinaka et al. 1985a, ten Dam et al. 1990, Wills et al. 1991, Goff 2004). Thus, in MLV and FeLV, the amber stop codon (UAG) at times is incorrectly read as glutamine (CAG) by the tRNA, causing the desired termination codon suppression (Yoshinaka et al. 1985b). Ribosomal or translational frameshifting involves the ribosome shifting 1 nt backwards (−1), as illustrated in Fig. 10. This (−1) shift allows the translation of different proteins from the same native mRNA, as demonstrated in FIV, HIV-1, RSV, and MMTV (Figs 10 and 2A) (Jacks and Varmus 1985, Jacks et al. 1987, 1988, ten Dam et al. 1990, Morikawa and Bishop 1992). For example, in the case of HIV-1 and other lentiviruses such as FIV, the (−1) frameshift happens only once before the Gag termination codon (Figs 10A and 2A). This shift changes the ORF from encoding the Gag precursor protein to encoding the Gag–Pol polyprotein (Jacks et al. 1988, Morikawa and Bishop 1992). However, in MMTV, HTLV-1, HTLV-2, simian retrovirus-1 (SRV-1), and MPMV, the (−1) frameshift happens twice at different junctions (Figs 10B and 2A). The first frameshift occurs before the Gag termination codon, changing the ORF from encoding the Gag precursor protein to encoding the Gag–Pro polyprotein. The second frameshift occurs before the Pro termination codon, changing the ORF from encoding the Gag–Pro precursor polyprotein to encoding the Gag–Pro–Pol polyprotein (Fig. 10B) (Jacks et al. 1987, Falk et al. 1993, Nam et al. 1993, ten Dam et al. 1994, Huang et al. 2013).

Mechanistically, ribosomal frameshifting relies on two critical components: a “heptameric slippery sequence” at the ribosomal frameshift site and a “pseudoknot” presented in the form of a complex RNA secondary structure present downstream of the frameshift signal (Fig. 10) (Chamorro et al. 1992). Typically, slippery sequences follow the X XXY YYZ pattern. For instance, the slippery sequence in HIV-1 is U_1_ UUU_4_ UUA_7_ (Jacks et al. 1988). As expected, ribosomal frameshifting in MMTV, HTLV-2, and MPMV relies on two such sequences (Fig. 10B). The first frameshift between Gag and Pro in MMTV, HTLV-1, and HTLV-2 follows the slippery sequence pattern of A AAA AAC, similar to HIV-1, while in MPMV, it is G GGA AAC (ten Dam et al. 1994, Huang et al. 2013). The second proposed slippery sequence for the frameshift between Pro and Pol in MMTV deviates from the HIV-1 pattern and consists of a tetrameric sequence, UUUA (Fig. 10B) (Jacks et al. 1987).

The pseudoknot facilitates the pausing of the ribosome, causing tRNA slippage. In the case of MMTV, FIV, MPMV, and SRV-1, the pseudoknot adopts a more intricate structure, indicating that the mechanism involves more than just ribosome stalling (Chamorro et al. 1992). For example, in the case of MMTV, unpaired A residues in the loop of the pseudoknot have also been implicated, which minimizes the coaxial stacking of helices, resulting in the frameshift event (Jacks et al. 1987, Chen et al. 1995b). Recently, in the case of RSV, a dynamic double pseudoknot junction has been reported, and it has been postulated that pseudoknot’s conformational heterogeneity plays an important role in significantly augmenting the frameshifting process (Jones and Ferré-D’Amaré 2025).

Ribosomal frameshifting in retroviruses is responsible for maintaining the stoichiometric ratio between viral proteins that are important for successful replication. It enables the expression of Gag and Gag–Pol at a ratio of 20:1 (Jacks et al. 1988, Shehu-Xhilaga et al. 2001). This ratio is consistent with the large number of Gag molecules required as precursors for structural proteins during virus assembly, while proteins such as PR, RT, and IN are needed in smaller quantities to perform their enzymatic and catalytic functions. Consistent with this, in the case of HIV-1, it has been documented that a 20:1 ratio of Gag versus Pol is essential for virus replication; any perturbation in this ratio is not tolerated by the virus, resulting in defects in replication, virion formation, and infectivity (Park and Morrow 1991, Karacostas et al. 1993, Shehu-Xhilaga et al. 2001, Biswas et al. 2004). This suggests that the expression levels of Gag and Gag–Pol must be precisely regulated to maintain a specific balance during virus assembly. Further evidence of this regulation comes from studies showing that perturbing ribosomal frameshift regulation or attempting to overexpress Gag–Pol in *trans* (as opposed to in *cis*) results in improper incorporation of Gag–Pol into virus particles. This negatively affects both the CA maturation process and the stability of the packaged dimeric RNA genome (Shehu-Xhilaga et al. 2001, Haraguchi et al. 2010, Yu et al. 2020, Benner et al. 2022). The critical reliance of HIV-1 on the ribosomal frameshift signal has garnered interest in targeting the frameshift mechanism for developing new classes of antiretrovirals (Biswas et al. 2004).

Ever since the discovery of internal ribosome entry site (IRES) in picornaviruses, its role in the translation of other virus groups, including retroviruses, has been well documented (Jang et al. 1988, Pelletier et al. 1988, Mailliot and Martin 2018). The role of IRES in retroviruses was first documented by the Darlix group, who investigated how the gRNA of MLV could produce two proteins from the Gag ORF. One protein was Pr65^Gag^, a precursor for structural proteins of the virus particle, while the other was the longer glycosylated isoform, Pr75^glyco-Gag^, which was presented on the surface of MLV-infected cells and exhibited mitogenic properties (Evans et al. 1977, Ledbetter and Nowinski 1977, Edwards and Fan 1979, Saris et al. 1983, Prats et al. 1989). In their seminal manuscript, Berlioz and Darlix show that an IRES mechanism promoted the translation of both Pr65^Gag^ and Pr75^glyco-Gag^ precursors (Berlioz and Darlix 1995). IRES elements have also been reported both in the 5′ UTR as well as in the Gag ORF of the primate *lentiviruses* (Ohlmann et al. 2000, Buck et al. 2001, Brasey et al. 2003, Nicholson et al. 2006, Weill et al. 2010). Even though the precise role of IRES in translation initiation among primate *lentiviruses* has yet to be fully established, it has been well documented that the presence of IRES in HIV-1, HIV-2, and SIV results in the translation of their N-terminally truncated Gag isoforms and both cap- and IRES-mediated mechanisms are used by the HIV-1 mRNA to drive the synthesis of the viral Gag protein (Buck et al. 2001, Herbreteau et al. 2005, Nicholson et al. 2006, de Breyne et al. 2012, 2013, Deforges et al. 2017).

The translation of envelope polyproteins in retroviruses is much less complex compared to the translation of Gag–Pol or Gag–Pro–Pol polyproteins. All retroviruses translate the envelope polyprotein from a singly spliced subgenomic mRNA, which is produced through a splicing event from the full-length viral RNA.

### Posttranslational modifications of retroviral proteins

Protein posttranslational modifications (PTMs) dramatically enhance the functional diversity of the proteome through various covalent modifications, including the addition of functional groups, proteolytic cleavage, disulfide bond formation, and so on. The addition of functional groups on proteins varies considerably such as, phosphorylation, glycosylation, ubiquitination, nitrosylation, methylation, acetylation, myristoylation, and so on. These modifications play crucial roles in controlling protein stability, altering cellular localization, modulating protein activity, and governing protein interactions, affecting a diverse array of cellular functions in eukaryotic cells. In the case of retroviruses, they offer mechanisms to transform an inhospitable cellular environment into a welcoming one, allowing the virus to continue its life cycle.

The most common PTM among retroviruses is myristoylation of Gag proteins which takes place in almost all known retroviruses except RSV, EIAV and foamy viruses (Rein et al. 1986, Copeland et al. 1988, Schultz and Rein 1989, Bryant and Ratner 1990, Weaver and Panganiban 1990, Bussienne et al. 2021). Myristoylation occurs cotranslationally and involves attachment of myristic acid (a saturated fatty acid) to the N-terminal glycine (Gly) of a protein (Jiang et al. 2018, Meinnel et al. 2020, Bussienne et al. 2021). This reaction is catalysed by *N*-myristoyltransferase, which is found exclusively in eukaryotes (Towler et al. 1987, Meinnel et al. 2020). One of the best studied consequences of myristoylation for virus replication is observed for the HIV-1 MA protein, where the “myristoyl switch” is important for regulation of Gag binding to the plasma membrane. This binding is dependent on the extent of exposure of the myristate moiety. MA trimerization exposes the myristic acid moiety on the Gag polyprotein, facilitating its anchoring to the plasma membrane to initiate virion particle assembly (Massiah et al. 1994, Hill et al. 1996, Zhou and Resh 1996, Spearman et al. 1997, Ono and Freed 1999). The other commonly occurring PTM (mostly studied in HIV-1) is the phosphorylation of Gag, which has been shown to modulate the incorporation of Vpr into virions to enhance infectivity (Kudoh et al. 2014). Furthermore, differential phosphorylation of the CA domain of Gag has been shown to affect important functions of the HIV-1 life cycle, including regulation of CA stability, assembly, uncoating and cDNA synthesis (Yu et al. 1995, Cartier et al. 1999, Kaushik and Ratner 2004, Takeuchi et al. 2017).

Retroviral envelope protein processing is similar to that of cellular membrane and secretory proteins, undergoing various PTMs that are critical for their functionality and infectivity of the virus. For instance, glycosylation of the envelope protein is one of the most important PTMs in retroviruses. This process occurs cotranslationally, during which a carbohydrate (or glycan) is covalently attached to the protein (Schwarz and Aebi 2011). Such a PTM not only ensures the structural integrity and proper localization of the envelope protein but also modulates its interactions with the host cell receptors, thereby enhancing virus’s ability to infect and replicate within the host (Walker et al. 1987, Fennie and Lasky 1989, Pal et al. 1989, Li et al. 1993). For an in-depth review of how specific PTMs affect the entire retroviral life cycle, see (Chen et al. 2018).

### Virus particle assembly

Retroviral particle assembly is a crucial phase in the viral life cycle, involving a series of highly orchestrated events that lead to the formation of infectious virions. This process begins with the translation of viral proteins and packaging of the viral RNA genome, facilitated by the structural Gag polyprotein, which plays a central role in driving virion assembly and release (Sundquist and Kräusslich 2012). The retroviral Gag polyproteins serve as the fundamental building block for retroviral particle assembly, often referred to as an “assembly machine,” where each assembling virus particle may contain as many as 2000–5000 Gag molecules (Wills et al. 1991, Vogt and Simon 1999, Briggs et al. 2004, Carlson et al. 2008, Chen et al. 2009). Consistent with this, the Gag proteins are both necessary and sufficient for the assembly of noninfectious virus-like particles (VLPs) in eukaryotic cells, which are capable of packaging the viral RNA and release from the host cell (Campbell and Rein 1999, Chameettachal et al. 2018, Pitchai et al. 2018, Krishnan et al. 2019, Pillai et al. 2023). In addition, purified Gag from prokaryotic cells can also assemble *in vitro* to form VLPs in the presence of yeast tRNA. These VLPs are morphologically identical to the immature virions produced by infected cells, though they are smaller in size than the 100–120 nm immature particles observed (Campbell and Vogt 1995, Klikova et al. 1995, Sakalian et al. 1996, Larson et al. 2005, Affranchino and González 2010, McKinstry et al. 2014, Tanwar et al. 2017, Chameettachal et al. 2018, Pitchai et al. 2018, Krishnan et al. 2019, Pillai et al. 2023).

Interestingly, in the case of HIV-1, the size restriction of the *in vitro* assembled VLPs could be overcome by adding inositol phosphates or phosphatidylinositol phosphates to the *in vitro* assembly reaction (Campbell et al. 2001). Careful functional, genetic, biochemical, and structural work conducted over the last decade has now made it abundantly clear that inositol hexakisphosphate (IP6) and others (like pentakisphosphate, IP5) play a crucial role in the assembly of not only HIV-1, but other retroviruses, such as RSV, EIAV, and MLV (Dick et al. 2018a, b, 2020, Obr et al. 2021, Biswas et al. 2024). The requirement of IP6/IP5 appears to be an essential conserved feature of retroviral replication, acting as general small-molecule cofactors during retrovirus assembly and maturation. More specifically, IP6/IP5 sit in a central pocket formed by a hexamer of CA and SP1 Gag peptides, enhancing Gag polymerization and assembly to ensure proper maturation, core stability, and increased virion yield.

Besides its key role in VLP formation, the Gag polyprotein is crucial for directing assembly to the plasma membrane in type C retroviruses (*alpha*-, *gamma*-, and *lentiviruses*; (Fig. 5). Especially in the case of HIV-1 myristoylation (myr) of MA is a prerequisite for Gag to bind to membranes (Bryant and Ratner 1990, Zhou and Resh 1996, Spearman et al. 1997, Nguyen and Hildreth 2000, Provitera et al. 2006). The fact that HIV-1 Gag tends to bind to membranes more effectively than the purified MA could be explained by the myristol switch mechanism, during which the myristol group is exposed in the context of full-length Gag, but gets sequestered in the purified MA protein (Zhou and Resh 1996, Spearman et al. 1997, Hermida-Matsumoto and Resh 1999, Ono and Freed 1999, Paillart and Göttlinger 1999). During Gag binding to the membrane, the MA domain remains attached to the inner face of the viral membrane, guiding the Gag or viral core to the plasma membrane and incorporating the envelope during virus assembly (Freed 1998). The association of MA with the plasma membrane is mediated by a highly basic region (HBR) and a hydrophobic myristic acid residue, collectively known as the “membrane binding domain” or M-domain (Ono et al. 2000, Chukkapalli et al. 2010). Later it became apparent that Gag localization to the plasma membrane is contingent on interactions between the HBR and phosphatidylinositol 4,5-bisphosphate (PIP_2_) (Ono et al. 2004, Chukkapalli et al. 2008, Chukkapalli and Ono 2011, Dick et al. 2012). Recent experiments using live cell imaging have shown that the HIV-1 assembly site heavily relies on (PIP_2_), that (PIP_2_) mobility in the cells is restricted by Gag, and Gag preferentially binds to membrane regions rich in phosphoinositide, sphingolipids, and cholesterol, known as lipid rafts (Mücksch et al. 2017, Favard et al. 2019, Tomishige et al. 2023). When MA binds to (PIP_2_) in these areas, it causes the myristic acid to insert into the membrane, which facilitates anchoring of Gag to the membrane (Ono et al. 2004, Saad et al. 2006, Waheed and Freed 2010, Freed 2015, Wen et al. 2020).

Besides HIV-1, the dependence of Gag assembly on PIP_2_ has also been observed in several other retroviruses, including MPMV; MLV, ASV, FIV, and HIV-2 (Stansell et al. 2007, Saad et al. 2008, Hamard-Peron et al. 2010, Prchal et al. 2012, Brown et al. 2015, Watanabe et al. 2018); however, it is the best studied in HIV-1. In HIV-1, (PIP_2_) is roughly three times more concentrated in the viral membrane compared to the plasma membrane and virus particles harbor three times more PIP2 molecules than Gag molecules (Chan et al. 2008, Mücksch et al. 2019). Furthermore, it has been suggested that (PIP2) functions both as an initiator of the myristoyl switch and a membrane anchor, potentially playing a key role in directing Gag to membrane rafts (Saad et al. 2006). Experiments targeted toward depleting (PIP_2_) in cells have shown Gag localization to intracellular membranes instead of the plasma membrane and severely compromised virus release, further validating the crucial role of (PIP_2_) in HIV-1 assembly (Ono et al. 2004, Chukkapalli et al. 2008).

Finally, MA multimerization (trimerization in the case of HIV-1) is crucial to expose the myristic acid that anchors Gag to the plasma membrane (Hill et al. 1996). Many other retroviruses, including SIV, EIAV, HTLV-2, BLV, and MPMV, exhibit MA trimers despite their highly varied sequences. Mutations in the trimer interface, at least in the case of MPMV, have been shown to compromise CA assembly (Rhee and Hunter 1991, Rao et al. 1995, Christensen et al. 1996, Matthews et al. 1996, Conte et al. 1997). In contrast, RSV Gag lacks myristoylation, and while MA multimerization aids in the electrostatic interaction with the plasma membrane, it may not be strictly needed for (PIP_2_) binding (Dalton et al. 2007).

The self-association of HIV-1 MA, and especially its trimerization, has also been shown to be crucial for the incorporation of envelope glycoprotein into the nascently forming virus particles (Dorfman et al. 1994, Freed and Martin 1995, Cosson 1996, Cannon et al. 1997, Tedbury et al. 2016, Eastep et al. 2021). The MA domain of Gag in other retroviruses has also been shown to be involved in envelope incorporation. For example, in RSV, MA and envelope chemically cross-link, while mutations in MPMV MA lead to inefficient envelope incorporation and postbudding cleavage of the envelope (Gebhardt et al. 1984, Rhee and Hunter 1990, Brody et al. 1992).

#### Role of gag domains in virion assembly

**Figure 11.**
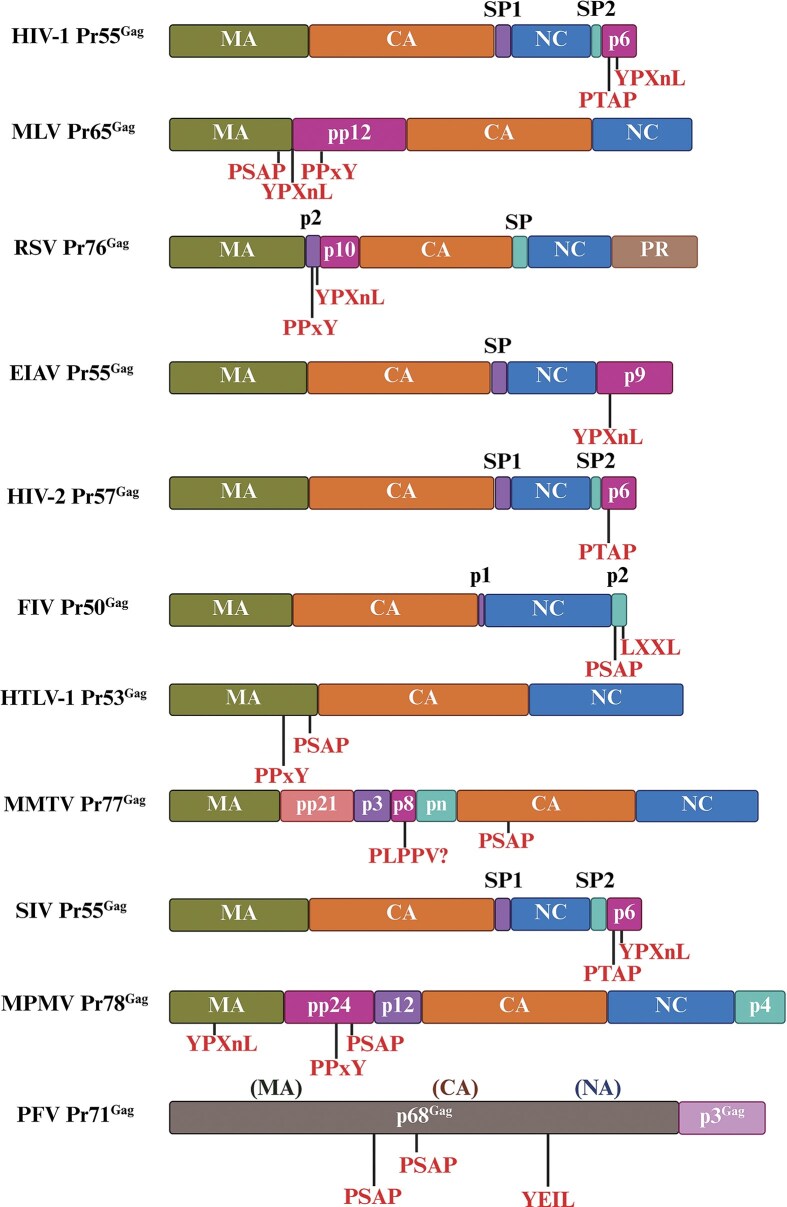
Illustration of the Gag domain organization in different retroviruses that include MA, CA, and NC. Spacer peptides (SP), other peptides (p or pp followed by a number), and late (L) domain-containing sequences are also shown. HIV/FIV/SIV, human, feline, and simian immunodeficiency viruses; MLV, murine leukemia virus; MMTV, mouse mammary tumor virus; and MPMV, Mason–Pfizer monkey virus. The L domain amino acids are as follows: PTAP: Pro-Thr-Ala-Pro; YPXnL (Tyr-Pro-any amino acid and n = ∼ 1–3 residues-Leu); PSAP: Pro-Ser-Ala-Pro; LXXL: Leu-any amino acid-any amino acid-Leu; PPxY: Pro-Pro-any amino acid-Tyr; and PLPPV: Pro-Leu-Pro-Pro-Val. Figure made in BioRender.com.

Figure 11 compares the domain structure of Gag proteins among retroviruses belonging to different genera. As can be seen, the order of the major Gag proteins, MA, CA, and NC, remains consistent among retroviruses; however, these domains are separated by a number of peptides and “spacer peptides (SP)”—short sequences of “linker” amino acids, the number and location of which varies between Gag proteins from different retroviruses (Fig. 11). These peptides and spacer peptides have important roles in virion assembly to ensure the generation of “infectious” virus particles. They achieve this by regulating the timing of Gag polyprotein processing and establishing correct virion morphology/core condensate structures within the virion, as well as ensuring proper virion budding from the host cell membrane (Wills and Craven 1991, Hunter 1994, Swanstrom and Wills 1997, Vogt 1997, Freed 1998, Bell and Lever 2013).

As mentioned earlier, retroviruses display different modes of virion assembly that can be either at the plasma membrane level (e.g. type C) or intracellularly within the cytoplasm, as exemplified by type B/D retroviruses (Fig. 5). The reason why some retroviruses follow the type-C assembly mode while others adopt the type-B/D assembly mode remain largely unclear. However, it is well-known that the key factors driving retroviral assembly are encoded within their Gag polyprotein. Even in the absence of other viral components, especially PR, expressing MMTV, FIV, and SIV Gag polyproteins independently directs the virus to the appropriate assembly pathway (Zábranský et al. 2009, Krishnan et al. 2019, Pillai et al. 2023). The critical role of Gag in virion assembly has been clearly demonstrated by amino acid substitutions(s) in the MA sequence of MPMV, MMTV, JSRV, and *spumaviruses*, resulting in the relocation of their assembly from cytoplasm to the plasma membrane—a major shift from a type D to a type C pattern (Rhee and Hunter 1990, Murcia et al. 2007, Zhang et al. 2015). The MA domain of these retroviruses includes a “cytoplasmic targeting and retention signal (CTRS)”, which is essential for assembling virus particles within the pericentriolar region of the cytoplasm. Amino acid change in the CTRS can shift CA assembly from the cytoplasm to the plasma membrane, highlighting the critical role of Gag in both VLP formation and the precise intracellular positioning of retrovirus assembly (Choi et al. 1999, Sfakianos et al. 2003, Yu et al. 2006). Along the same lines, alterations in the MA domain of HIV-1 Gag, whether through amino acid substitutions or deletions, have been shown to redirect virus assembly to the cytoplasm or intracellular membranes, such as the endoplasmic reticulum (Fäcke et al. 1993, Cannon et al. 1997). These observations suggest that, despite differences in assembly modes, the processes governed by Gag across various retroviruses may be more similar than previously assumed (Junková et al. 2020).

The process of Gag multimerization is essential for virus assembly, relying on both Gag–Gag and Gag–gRNA interactions. The primary region responsible for Gag multimerization is the C-terminal part of the CA domain (Freed 2015). Additionally, basic residues within the NC domain are believed to play a crucial role in Gag multimerization, which is mediated by the interaction domain (I-domain) (Dawson and Yu 1998, Freed 2015). Furthermore, for efficient CA–CA interactions, gRNA binding to CA is necessary so that I-domains within CA can be readily exposed (Burniston et al. 1999, Ott et al. 2009, Tanwar et al. 2017, Yang et al. 2018). This results in oligomerization of HIV-1 Gag on gRNA and its transport to the plasma membrane, where further multimerization occurs (Yang et al. 2018), indicating that gRNA capture and/or packaging and virus assembly are closely linked processes.

#### gRNA packaging

**Figure 12.**
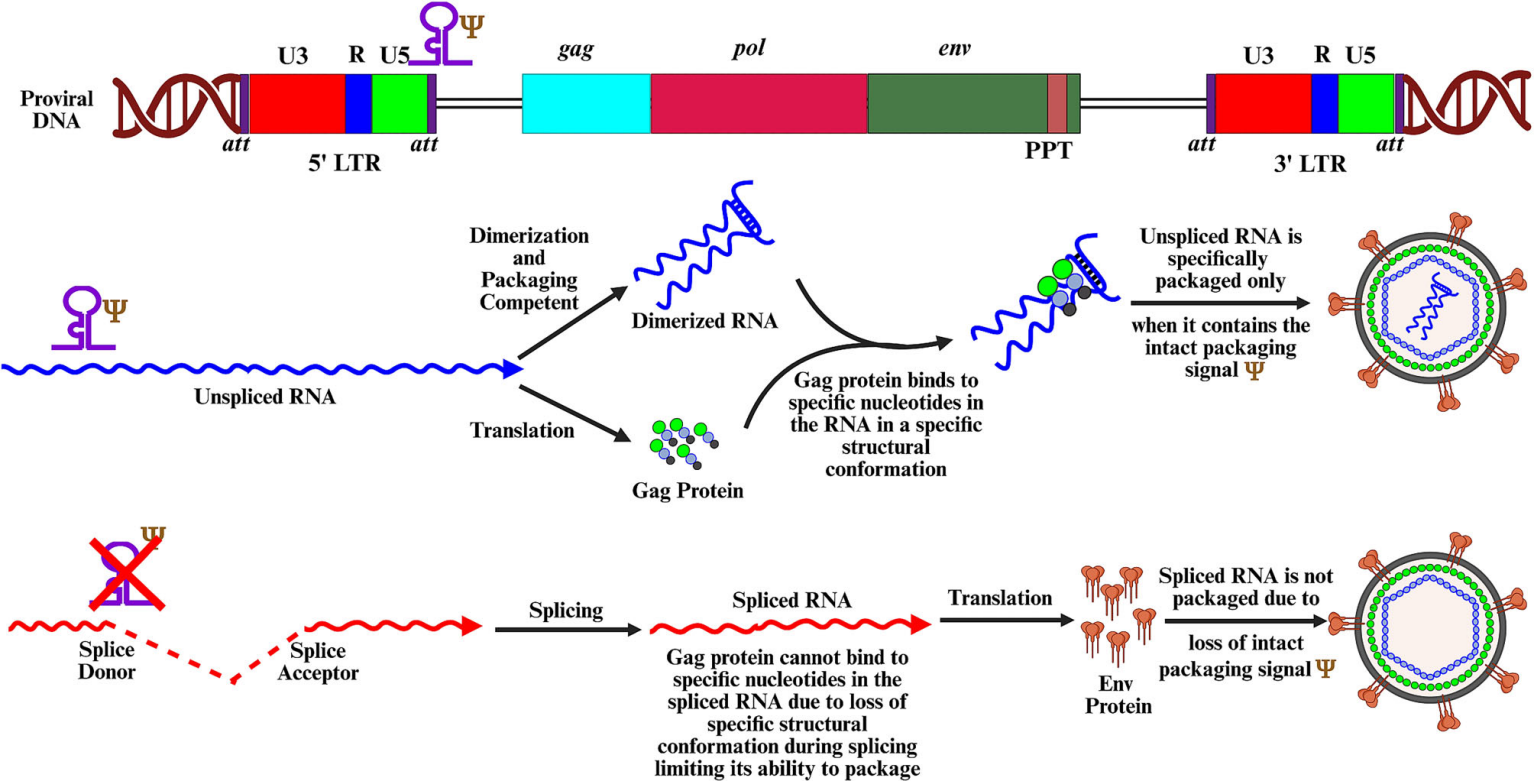
A simplistic model of retroviral gRNA packaging based on selective recognition of the viral genome by the structural Gag polyprotein based on the recognition of the higher-order structure of the *cis*-acting packaging sequences (*Psi*: ψ). Following transcription from the proviral DNA, unspliced and spliced RNAs are generated which have different fates as illustrated in the figure. Figure made in BioRender.com.

Retroviral genome packaging is a highly selective process that ensures the encapsidation of two full-length, unspliced RNA genomes into assembling virions. This takes place with great specificity despite being present in low abundance in the infected cells in comparison to host and viral subgenomic RNAs (D’Souza and Summers 2005, Lever 2007, Rulli et al. 2007, Johnson and Telesnitsky 2010, Miyazaki et al. 2011, Comas-Garcia et al. 2016, Maldonado and Parent 2016). This process is tightly coordinated with virus assembly, driven primarily by the Gag precursor polyprotein, which recognizes structural motifs within the 5′ UTR and adjacent *gag* sequences, collectively referred to as the packaging signal (*Psi*, Ψ; Fig. 12) (Berkhout and van Wamel 1996, Das et al. 1997, Clever et al. 2002, D’Souza and Summers 2005, Lever 2007, Rulli et al. 2007, Johnson and Telesnitsky 2010, Jouvenet et al. 2011a, Miyazaki et al. 2011, Comas-Garcia et al. 2016, Mailler et al. 2016, Maldonado and Parent 2016).

Structure–function studies across diverse retroviruses, including HIV-1, MMTV, MPMV, and FIV, have consistently demonstrated that RNA packaging elements form complex secondary and tertiary structures. These structured motifs often include stem-loops, bulges, and unpaired purine-rich sequences that serve as high-affinity Gag binding sites (Clever et al. 1995, D’Souza and Summers 2004, 2005, Lever 2007, Johnson and Telesnitsky 2010, Didierlaurent et al. 2011, Abd El-Wahab et al. 2014, Comas-Garcia et al. 2016, Bernacchi et al. 2017, Johnson 2017, Chameettachal et al. 2021, Pitchai et al. 2021, Krishnan et al. 2023, Prabhu et al. 2024). For instance, in HIV-1, HIV-2, HTLV-1, SIV, MMTV, and MPMV, single-stranded purine or unpaired stretches are critical for gRNA packaging, with mutational disruption leading to impaired Gag binding and gRNA packaging (De Guzman et al. 1998, Baig et al. 2009, Abd El-Wahab et al. 2014, Keane et al. 2015, Keane and Summers 2016, Comas-Garcia et al. 2018, Singh et al. 2018, Wu et al. 2018, Ali et al. 2020, Ding et al. 2020, Nikolaitchik et al. 2020, Chameettachal et al. 2021, Pillai et al. 2021, Pitchai et al. 2021, Umunnakwe et al. 2021). The Gag polyprotein first selectively recognizes gRNA amongst a variety of cellular and spliced viral RNAs, and such RNA–Gag binding favors Gag multimerization (Didierlaurent et al. 2011, Abd El-Wahab et al. 2014, Bernacchi et al. 2017, Pitchai et al. 2021).

Over the years it has now become clear that the selectivity by which Gag binds to specific nucleotides in the packaging sequences is largely governed by RNA secondary structural motifs rather than primary sequence, as schematically shown in Fig. 12. The preference for packaging full-length, unspliced RNA over its spliced counterparts can be explained based on the RNA secondary structure of the packaging sequences. For instance, splicing can disrupt key packaging sequences by removing them or altering their structure. This causes Gag-binding nucleotides to become base-paired and inaccessible thereby impairing Gag’s ability to recognize and bind to these nucleotides in the RNA, ultimately limiting its dimerization and packaging abilities during viral assembly. Such structural occlusions have recently been observed in spliced RNAs of MMTV, MPMV, and FIV (Chameettachal et al. 2021, Pitchai et al. 2021, Krishnan et al. 2023).

Initially it was thought that the NC domain of Gag is the key player during selective retroviral gRNA packaging, primarily owing to its basic charge and the presence of Cys-His boxes having strong affinity to interact with Zn^2+^ ions that augmented protein/RNA interactions (Gorelick et al. 1988, Aldovini and Young 1990, Wang and Barklis 1993, Lever 2007, Lu et al. 2011, Ali et al. 2016, Maldonado and Parent 2016, Rawson et al. 2022). However later studies, especially focusing on HIV-1, demonstrated that full-length Gag has higher binding specificity than NC alone, suggesting that multiple Gag domains may be involved in contributing to gRNA recognition (Gherghe et al. 2010). Consistent with this, early focus on HIV-1 stem-loop 3 (SL3) as being the major packaging determinant was revisited when the full-length Gag was shown to preferentially bind to a G//AGG loop in SL1, rather than the GGAG tetraloop in SL3 (De Guzman et al. 1998, Abd El-Wahab et al. 2014, Keane et al. 2015, Bernacchi et al. 2017, Ding et al. 2020).

Dimerization of gRNA is another conserved feature that links gRNA packaging to viral recombination and replication fidelity (Berkhout and van Wamel 1996, Mougel et al. 1996, Paillart et al. 1996a, b, McBride et al. 1997, Mougel and Barklis 1997, Mikkelsen et al. 2000, Parent et al. 2000, Aagaard et al. 2004, Chin et al. 2007, Houzet et al. 2007, Moore et al. 2007, 2009, Sakuragi et al. 2007, Onafuwa-Nuga and Telesnitsky 2009, Johnson and Telesnitsky 2010, Dubois et al. 2018). The dimerization starts at the dimerization initiation site (DIS), which is typically located in the 5′ leader region within the packaging sequences and harbors a conserved “GC” dyad, enabling two RNA genomes to form a noncovalent dimer via kissing-loop interactions (Laughrea and Jetté 1994, Paillart et al. 1994, Skripkin et al. 1994, Clever et al. 1996, Polge et al. 2000, Aktar et al. 2013, 2014). In HIV-1, disruption of the DIS abolishes dimerization and impairs gRNA packaging (Skripkin et al. 1994, Berkhout and van Wamel 1996, Clever et al. 1996, Paillart et al. 1996a, 1997, Laughrea et al. 1997). In MMTV and MPMV, palindromic sequences containing canonical “GC” dyads similarly mediate dimer formation, and their mutation drastically reduces dimerization and viral replication (Aktar et al. 2013, 2014). Furthermore, in the case of MLV, following dimerization, the UCUG motif, critical for Gag binding, becomes exposed (which is otherwise sequestered in the monomeric RNA), further linking RNA structure to genome selection during gRNA packaging (D’Souza and Summers 2004). Thus, viral assembly revolves around a gRNA dimer that then requires the Gag selectivity switch from specific to nonspecific RNA binding (Kutluay et al. 2014).

Long-range intramolecular RNA–RNA interactions (LRIs) also contribute significantly to RNA structure and packaging specificity. For example, in the case of HIV-1, the packaging sequences adopt two alternative conformations: a translationally active long-distance interaction and a “packageable” branched multiple hairpin form where the DIS and Gag-binding sites are exposed (Huthoff and Berkhout 2001, Abbink and Berkhout 2003, Abbink et al. 2005, Keane et al. 2015). Similar LRI-mediated RNA rearrangements have been observed in MPMV, FIV, and MMTV where complementary interactions between U5 and downstream sequences stabilize the overall RNA secondary structure facilitating gRNA packaging (Kalloush et al. 2016, Krishnan et al. 2023, Prabhu et al. 2024). Furthermore, in the case of MMTV, disrupting U5–U5 LRIs has been shown to abolish gRNA packaging despite preserving Gag-binding elsewhere, indicating that packaging requires specific RNA structures in the correct three-dimensional context for Gag to bind to the primary binding sites (Prabhu et al. 2024).

Additional regulation for gRNA packaging comes from transcriptional start site heterogeneity and epitranscriptomic modifications. HIV-1 generates multiple unspliced RNA species (e.g. 1 G versus 3 G RNAs) that differ only slightly at the 5′ end, but fold into distinct structures with differing packaging efficiencies. The 1 G RNA preferentially adopts conformations that leave the DIS and Gag-binding motifs such as unpaired purines fully exposed thus favoring gRNA packaging. On the other hand, the 3 G RNA adopts a different conformation in which DIS and Gag binding sites are obscured, rendering it more disposed to translation than to packaging (Masuda et al. 2015, Kharytonchyk et al. 2016, Brown et al. 2020, Ding et al. 2021, Nikolaitchik et al. 2023). Similarly, RNA modifications, particularly N6-methyladenosine (m^6^A), impact gRNA fate. It has been shown that demethylated RNA is more efficiently packaged, while the methylated form (m^6^A-marked RNA) supports translation (Pereira-Montecinos et al. 2022, Singh et al. 2022b, Baek et al. 2024).

In light of the above observations, it is fair to conclude that retroviral gRNA packaging is a structurally driven, multifaceted process regulated by Gag recognition of conserved RNA structural motifs, dimerization signals, and long-range interactions. Understanding the nuances of this process remains essential for uncovering general principles of retroviral replication and well thought out therapeutic strategies.

### Budding or release, envelope incorporation, and maturation

Virus particle release is a crucial step in the retroviral life cycle, enabling newly assembled virions to exit the host cell to establish new infection and continuity of the viral life cycle. This process is driven by the viral structural polyprotein Gag, which directs particle assembly at the plasma membrane and facilitates membrane curvature to promote budding/release (Sundquist and Kräusslich 2012). To achieve efficient release, retroviruses hijack the host cell’s endosomal sorting complexes required for transport (ESCRT) machinery, a cellular system typically involved in membrane remodeling and vesicle trafficking (Wollert and Hurley 2010, Votteler and Sundquist 2013, Olmos and Carlton 2016, Schemelev et al. 2024). Retroviral Gag has been implicated in recruiting ESCRT machinery via conserved motifs, termed late assembly domains (“L” domains). These short, 4–6 amino acid domains act like cellular adaptor proteins, ensuring membrane scission and virion separation from the host cell membranes (Fig. 11) (Freed 2015, Welker et al. 2021). The “L” domains have been reported in many retroviruses, such as HIV, MLV, RSV, EIAV, HIV-2, FIV, HTLV-1, MPMV, and FV and are labeled in red in Fig. 11 (Wills et al. 1994, Parent et al. 1995, Puffer et al. 1997, Patnaik et al. 2000, Schubert et al. 2000, Strack et al. 2000, Vogt 2000, Le Blanc et al. 2002, Myers and Allen 2002, Gottwein et al. 2003, Patton et al. 2005, Narahara and Yasuda 2015, Bartusch and Prange 2016, Del Vecchio et al. 2020). Their location and number vary, and some are common among retroviruses, such as the PSAP, PTAP, PPxY, and YPXnL motifs, while others are unique, such as LXXL, PLPPV, and YEIL. In addition to the L domains, ubiquitination of Gag is a common feature among retroviruses, intricately associated with retroviral budding/release (Patnaik et al. 2000, Strack et al. 2000, Vogt 2000, VerPlank et al. 2001).

In case of HIV-1, the “L” domains (PTAP and YPXnL) are present in the C-terminally located p6 protein (52 amino acids), deletion of which severely affects virion release, leading to virions bound to the plasma membrane (Fig. 11) (Göttlinger et al. 1991). Mutational analysis of HIV-1 p6 has been instrumental in identifying a short Pro-Thr/Ser-Ala-Pro (PT/SAP) motif, which is critical for particle release (Huang et al. 1995, Demirov et al. 2002). HIV-1 exploits host proteins such as tumor susceptibility gene 101 (TSG101) and ALG-2 interacting protein X (ALIX) to enhance its budding/release. In particular, the PT/SAP motif recruits ESCRT-I subunit TSG101, while Tyr-Pro-Xn-Leu (YPXnL, where X is any residue and n is 1–3 amino acids) motif in the p6 mediates the interaction with ALIX, a component of ESCRT-III complex (Fig. 11) (Garrus et al. 2001, Martin-Serrano et al. 2001, VerPlank et al. 2001, Demirov et al. 2002, Strack et al. 2003, Fisher et al. 2007, Lee et al. 2007, Munshi et al. 2007, Zhai et al. 2008, Dussupt et al. 2009, Friedrich et al. 2016, Schemelev et al. 2024). Next, key ESCRT-III components, such as charged multivesicular body proteins 2 and 4 (CHMP2 and CHMP4), and the vacuolar protein sorting-associated protein 4 ATPase (VPS4 ATPase), are recruited to the budding site at the plasma membrane. These components assemble into spiral filaments on the inner membrane of the budding virion’s neck, facilitating the decisive intrinsic membrane separation necessary for the completion of virion release (Hanson et al. 2008, Jouvenet et al. 2011b, Morita et al. 2011, Shen et al. 2014). Curiously enough, a proposed model for HIV-1 release through the PT/SAP TSG101 pathway suggests that NC also collaborates with the PTAP motif in p6 to recruit essential cellular proteins. This process also involves interactions with Bro1-containing proteins and potentially other host factors necessary for YPXnL-mediated particle release (Dussupt et al. 2009). As can be seen, HIV-1 Gag p6 protein plays a crucial role in viral replication by mediating interactions with host factors essential for budding/release of particles. Thus, targeting Gag p6 represents a promising strategy for the development of next-generation therapeutics aimed at curbing HIV-1 replication by blocking this essential step in viral replication (Chen and Wang 2024).

Similarly, mutagenesis studies of RSV Gag identified two “L” domains (PPxY and YPXnL) located N-terminally between the MA and CA domains in the p2b spacer peptide essential for viral particle release (Fig. 11). Truncating p2b exerts severe budding defects, similar to those seen in HIV-1 p6 mutants (Göttlinger et al. 1991, Wills et al. 1994). Notably, RSV Gag chimeras demonstrated that the L domain function of p2b could be substituted by the C-terminal sequence of HIV-1 p6, regardless of its position in the precursor (Parent et al. 1995). Likewise, the “L” domain of the Ebola virus harbors PT/SAP and PPxY motifs within its VP40 protein, which can compensate for the absence of HIV-1 NC-p1 and p6 regions, facilitating HIV-1 particle release (Strack et al. 2002). These findings suggest that, despite structural differences, “L” domains of diverse viruses serve a common role in facilitating viral particle release.

The efficient trafficking and incorporation of the Env protein into retroviral particles is a tightly regulated process essential for viral infectivity. Env is synthesized in the endoplasmic reticulum where it undergoes extensive PTMs, including glycosylation and proteolytic cleavage, as it progresses through the Golgi apparatus before reaching the plasma membrane. In case of HIV-1, the Env glycoproteins precursor, gp160, is cleaved in the Golgi by cellular furin or furin-like PRs into: (i) a mature surface glycoprotein gp120 and (ii) the transmembrane glycoprotein gp41 (Willey et al. 1988, Stein and Engleman 1990, Hallenberger et al. 1992, Checkley et al. 2011). Host factors and cellular trafficking pathways, including the secretory and endocytic recycling systems, contribute to Env’s proper distribution and availability at sites of viral assembly (Anokhin and Spearman 2022). Proper localization of Env at the plasma membrane is critical for its selective incorporation into budding virions, a process mediated by interactions between gp41 cytoplasmic tail and the MA domain of the Gag protein (Dorfman et al. 1994, Freed et al. 1995, Cosson 1996, Freed and Martin 1996, Vincent et al. 1999, Murakami and Freed 2000). During viral assembly, three molecules of the gp120/gp41 complex are incorporated into the lipid bilayer of the nascently forming virions in the form of a trimer of heterotrimeric spikes. Dysregulation in any of the abovementioned pathways can significantly impair viral infectivity, highlighting the importance of precise Env trafficking and incorporation to the retroviral life cycle.

Once a retrovirus completes its assembly, it buds off as an immature, noninfectious virion containing viral components as large polyproteins, primarily Gag and Gag–Pol (such as MLV and HIV-1) assembling at the plasma membrane (Fig. 5), and Gag, Gag–Pro, and Gag–Pro–Pol (such as MPMV and MMTV), preassembling in the cytoplasm (Fig. 5) (Coffin 1997, Sundquist and Kräusslich 2012, Freed 2015, Pornillos and Ganser-Pornillos 2019, McGraw et al. 2024). However, for the virus to become infectious, these polyproteins must undergo precise cleavage and reorganization, a process known as “maturation.” Retroviral maturation is a meticulously orchestrated event that determines whether a virus can successfully propagate or not. Through intricately controlled enzymatic activity and structural remodeling, a once inert particle transforms into a formidable infectious agent, ready to perpetuate its life cycle. Therefore, retrovirus maturation can be viewed as the conversion of a membrane-bound immature particle into a freely diffusible mature core, where the viral genome becomes ready to initiate infection upon entering the host cell’s cytoplasm.

The process of maturation begins with the activation of the viral PR, an enzyme embedded within the Gag–Pol, Gag–Pro, or Gag–Pro–Pol precursor, depending upon the retrovirus type. Retroviral PRs are classified as aspartyl PRs and contain a conserved active site motif. In viruses such as HIV-1, HIV-2, SIV, FIV, and MMTV, this motif consists of the Asp-Thr-Gly (DTG) sequence, whereas in RSV, it is slightly modified to Asp-Ser-Gly (DSG) (Wlodawer and Gustchina 2000, Konvalinka et al. 2015). Initially inactive, the retroviral PR undergoes an autocatalytic activation upon budding, triggering a cascade of cleavage events that cleaves the Gag precursor polyprotein into the three main structural proteins, MA, CA, and NC (Coffin 1997, Sundquist and Kräusslich 2012, Freed 2015, Pornillos and Ganser-Pornillos 2019). Additional Gag cleavage products have also been reported, but these are not conserved across different retroviral genera (Fig. 11). The general organization of these Gag domains (MA, CA, and NC) is highly conserved among different retroviruses (Fig. 11) (Wills and Craven 1991, Hunter 1994, Swanstrom and Wills 1997, Vogt 1997, Freed 1998, Bell and Lever 2013). The MA domain remains associated with the inner face of the viral membrane and plays a crucial role in directing Gag or the viral core to the plasma membrane and incorporating the envelope during virus assembly (Freed 1998). The CA domain forms the building block of the viral core, condensing to create a shell around the viral RNA/NC complex. The NC domain, located within the core, is tightly associated with the retroviral gRNA, protecting it from degradation within the hostile environment of the cell (Méric et al. 1984). Gag is critical for the encapsidation of gRNA into the assembling virus particles, with the NC domain being the most relevant for RNA packaging (Kohl et al. 1988, Luban and Goff 1991, Berkowitz et al. 1993, Berkowitz and Goff 1994, Dannull et al. 1994, Clever et al. 1995, Geigenmüller and Linial 1996). Improper cleavage by the PR results in defective, noninfectious retroviral particles (Kohl et al. 1988, Coffin 1997, Sundquist and Kräusslich 2012, Könnyű et al. 2013, Freed 2015, Pornillos and Ganser-Pornillos 2019). Similarly, mutations in the PR coding domain or inhibition of PR activity result in virions containing unprocessed Gag, rendering them noninfectious (Crawford and Goff 1985, Katoh et al. 1985, Kohl et al. 1988, Ashorn et al. 1990, Meek et al. 1990, Roberts et al. 1990, Stewart et al. 1990, Sommerfelt et al. 1992).

**Figure 13.**
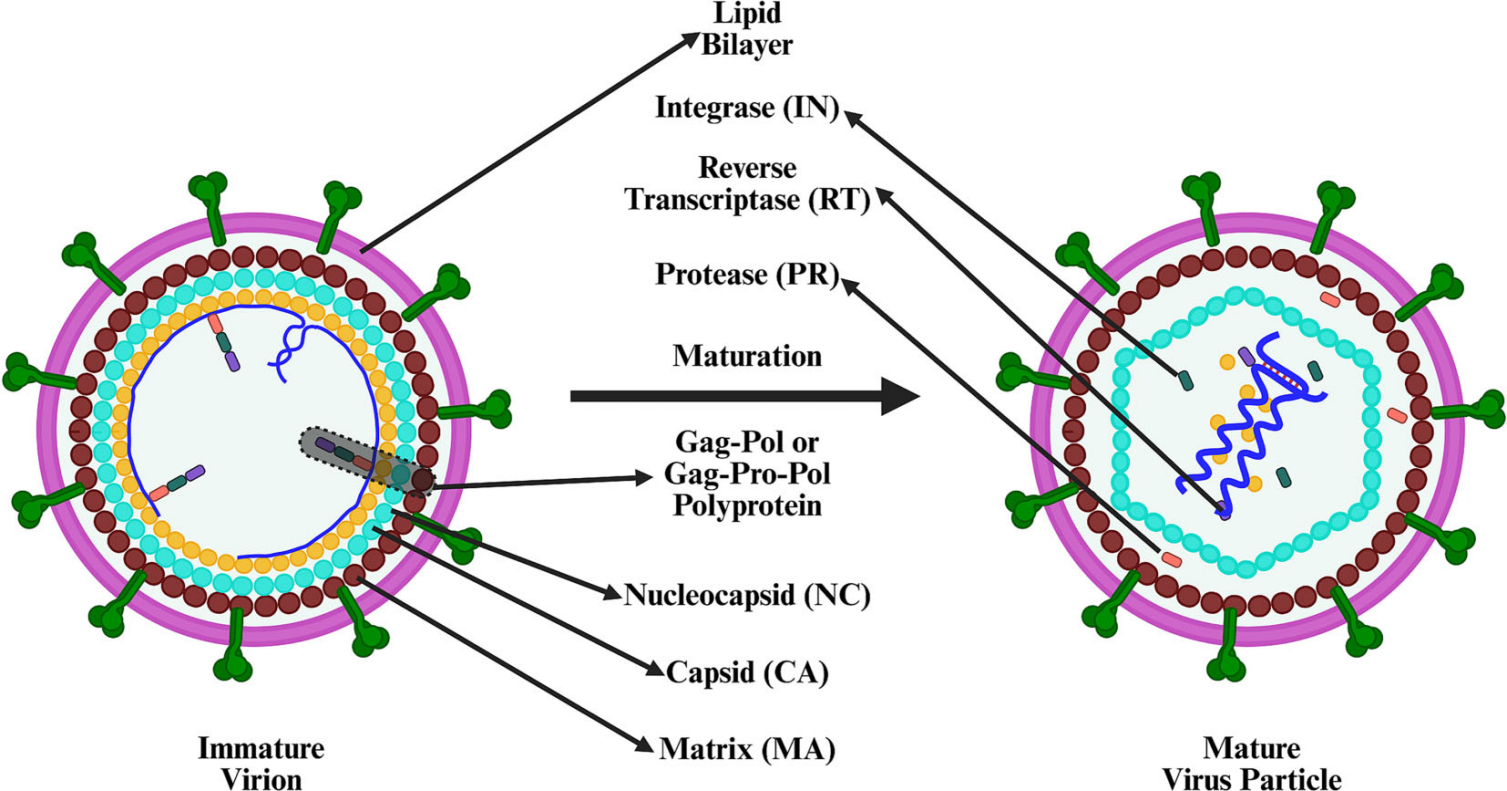
Comparison of an immature retroviral particle shortly after budding (left) and a mature particle (right) following activation of the viral PR. Figure made in BioRender.com.

Once the Gag polyprotein is processed, the structural rearrangement of the virion begins. In immature virions (Fig. 13), the Gag precursor remains in an amorphous lattice anchored to the inner surface of the viral envelope, whereas in the mature and processed virions, they assemble into an electron-dense, variably shaped cores, depending upon the virus type, which is a hallmark of mature retroviruses (Fig. 1) (Bolognesi et al. 1978, Gelderblom et al. 1987, Menéndez-Arias et al. 1992). Such structural differences have been observed in viruses that assemble at the plasma membrane, such MLV and HIV-1, as well as those that preassemble in the cytoplasm before budding, such as MPMV and MMTV (Luftig and Yoshinaka 1978, Katoh et al. 1985, Voynow and Coffin 1985, Göttlinger et al. 1989, Peng et al. 1989, Stewart et al. 1990, Sommerfelt et al. 1992). The core in mature retroviruses encases the viral RNA genome along with essential enzymes, such as RT and IN, ensuring that the virus is primed for infection (Fig. 13) (Pornillos and Ganser-Pornillos 2019). With the structural transformation complete, the virus achieves full infectivity. A mature virion can now bind to a new host cell, initiate fusion, and deliver its genetic material for replication. This understanding of maturation has been pivotal in antiretroviral therapy, leading to the development of PR inhibitors that effectively block HIV-1 progression (Adamson and Freed 2007, Adamson et al. 2009, Waki et al. 2012, McGraw et al. 2024).

## Perspectives/concluding remarks

Retroviruses represent a unique group of RNA viruses, whose replication strategy has not only challenged the central dogma of molecular biology, but has also revolutionized our understanding of molecular virology, viral evolution, and host–pathogen interactions. Their hallmark features such as reverse transcription followed by chromosomal integration have offered profound insights into gene expression, transcriptional regulation, and cellular transformation. Over the past several decades, retroviruses have served as invaluable tools in both fundamental and translational research, facilitating discoveries that laid the groundwork for modern molecular biology, including the identification of oncogenes, the development of recombinant DNA technology, and the advent of gene therapy.

This review has highlighted the multifaceted molecular strategies employed by retroviruses at each step of their life cycle, starting from receptor-mediated entry, integration of their proviral DNA into the host genome, to the orchestrated expression of viral genes. While foundational aspects such as reverse transcription and integration are now well understood, newer findings, particularly those pertaining to the timing and location of uncoating, nuclear entry of CAs, and the multifunctional roles of viral proteins, such as IN and Gag are reshaping our understanding of retroviral biology. For instance, recent evidence revealing nuclear reverse transcription and CA trafficking challenges long-standing dogmas and opens new avenues for therapeutic intervention. Similarly, the realization that integration site selection is not entirely random, but rather influenced by viral and host determinants, has important implications for both pathogenesis and gene therapy vector safety.

Despite the significant progress made, several outstanding questions remain. For example, the dynamics of proviral transcriptional regulation, and the contribution of host cell factors in modulating retroviral replication efficiency are areas open for further exploration. Additionally, with the increasing use of retroviral vectors in clinical settings, particularly for *ex vivo* gene therapy, a deeper understanding of vector–host interactions is essential to enhance efficacy and minimize risks such as insertional mutagenesis.

Looking ahead, integrative approaches, combining structural biology, high-resolution imaging, single-cell transcriptomics, and genome editing technologies will be vital in addressing these knowledge gaps. Such interdisciplinary efforts hold the potential not only to unravel some of the remaining outstanding questions of retroviral replication, but also to refine the therapeutic utility of retroviral systems. As our understanding of retroviruses continues to evolve, so too will be our ability to harness and combat them with implications reaching far beyond virology into cancer biology, immunology, and regenerative medicine.
